# Protection of mice against septic shock induced by lethal intraperitoneal dose of LPS by a recombinant *Fasciola hepatica* fatty acid binding protein

**DOI:** 10.1128/spectrum.02756-25

**Published:** 2026-02-27

**Authors:** Albersy Armina-Rodriguez, Stephanie M. Dorta-Estremera, Loyda B. Mendez, Ana M. Espino

**Affiliations:** 1 Department of Microbiology and Immunology, University of Puerto Rico-Medical Sciences Campus 12320, San Juan, Puerto Rico; 2 Division of Clinical and Translational Cancer Research, Comprehensive Cancer Center University of Puerto Rico 591857 , San Juan, Puerto Rico; 3 School of Science & Technology, University Ana G. Mendez, Carolina Campus 19860 , Carolina, Puerto Rico; Debreceni Egyetem, Debrecen, Hungary

**Keywords:** septic shock, *Fasciola hepatica*, fatty acid binding protein, inflammation

## Abstract

Sepsis caused by Gram-negative bacteria is a severe systemic infection characterized by an excessive inflammatory response triggered by lipopolysaccharide (LPS), a potent endotoxin of these bacteria, often leading to organ failure and high mortality. Despite improved outcomes with antibiotic therapy, controlling persistent inflammation remains a major therapeutic challenge. Interestingly, helminth-derived molecules, such as the recombinant protein Fh15 from *Fasciola hepatica*, have been demonstrated to suppress inflammatory responses in experimental models of sepsis. This study aimed to evaluate the capacity of Fh15 to effectively attenuate cytokine storms and improve survival in mice, alongside analyzing its effects on macrophage phenotype and function to better understand its potential as an anti-inflammatory biotherapeutic. Our results demonstrate that a single intraperitoneal injection of an optimized dose of 100 μg Fh15 (~5 mg/kg body weight), administered 1 h after a lethal (LD100) LPS challenge, significantly improved survival and markedly suppressed the cytokine storm in mice. This treatment was associated with a substantial reduction in C-reactive protein (CRP) levels, along with an increase in IL-37. In addition, a 100 μg Fh15 dose (~5 mg/kg body weight [BW]) effectively controlled LPS-induced IL-10 overproduction, potentially preventing immunosuppression that may contribute to mortality. Fh15 also significantly reduced the expression of macrophage activation markers, including CD14, tumor necrosis factor-alpha (TNF-α), nitric oxide synthase-2 (NOS2), CD38, and IL-12p70, potentially impairing macrophage maturation, phagocytic activity, and their capacity to activate Th1 cells. These findings demonstrate the potential of Fh15 to modulate key inflammatory pathways involved in sepsis and support its potential as a novel recombinant protein-based anti-inflammatory therapy.

**IMPORTANCE:**

Sepsis remains a leading cause of mortality worldwide, primarily driven by an overwhelming inflammatory response triggered by bacterial components such as lipopolysaccharide (LPS). This excessive immune activation, characterized by cytokine storms and macrophage overactivation, leads to organ failure and death. Current therapeutic strategies, including antibiotic treatment, often fail to address the persistent inflammation that contributes to sepsis severity. In this study, we proposed Fh15, a recombinant protein derived from *Fasciola hepatica*, as a potent immunomodulatory agent capable of attenuating the cytokine storm, improving survival, and modulating macrophage activity in a mouse model of septic shock. These findings underscore the potential of Fh15 as a novel anti-inflammatory agent, offering a promising therapeutic approach to manage endotoxemia and reduce sepsis-related mortality.

## INTRODUCTION

Sepsis caused by Gram-negative bacteria is a life-threatening condition resulting from an exaggerated inflammatory response to infection, with mortality rates that can be as high as 30% (1). Lipopolysaccharide (LPS), also known as endotoxin, is a major component of the outer membrane of Gram-negative bacteria and is responsible for triggering this extreme systemic response (2). When LPS is recognized by toll-like receptor-4 (TLR4) on innate immune cells, a nuclear factor-kappa B (NF-κB)-dependent inflammatory cascade is initiated. This cascade strongly influences classical macrophage (M1) activation. M1 polarization results in increased production of inflammatory cytokines, such as tumor necrosis factor-alpha (TNF-α) and interleukin-6 (IL-6), among others. These cytokines ultimately lead to endothelial cell damage, increased vascular permeability, low blood pressure, and organ failure (3). Although the use of antibiotics has improved the prognosis of sepsis, the persistently high mortality rate has been attributed to the uncontrolled inflammatory response. Therefore, neutralization of inflammation has become a key therapeutic approach to reduce the high mortality associated with severe sepsis (4).

Helminths are potent immunomodulatory parasites capable of triggering multiple mechanisms that protect immunocompetent mammals against inflammatory diseases, including sepsis (5). However, helminth infections are also highly pathogenic, capable of causing debilitating symptoms, malnutrition, and multiple physiological side effects that can compromise normal immune function. Consequently, it is more judicious to identify and characterize the specific immunomodulatory molecules produced by helminth parasites and to elucidate their precise mechanism of action.

Our laboratory has studied the interaction between antigens derived from the trematode parasite *Fasciola hepatica* and immune cells for over a decade. We have described a recombinant fatty acid-binding protein (FABP), termed Fh15, that exhibits potent anti-inflammatory properties. Using mouse and rhesus macaque models of sepsis and septic shock, we demonstrated that a single intraperitoneal (i.p.) injection of 50 μg Fh15 in mice or a single intravenous infusion of 12 mg Fh15 in rhesus macaques was enough to significantly suppress the cytokine storm and other inflammatory markers induced by lethal doses of LPS or live *Escherichia coli*, respectively (6, 7). However, it remains unknown whether the dose of Fh15 used is sufficient to maintain sustained suppression of the cytokine storm over time and to reduce mortality. Moreover, although our previous *in vitro* studies revealed that Fh15 suppresses the NF-κB activation and TNF-α production in macrophages stimulated with LPS (8), it is unclear whether these findings translate into direct modulation of macrophage activation and function.

In the present study, we performed a dose-response survival experiment to determine the dose of Fh15 that most efficiently suppresses the cytokine storm and most effectively increases the mouse survival rate over time. Additionally, we performed *in vitro* characterization of the effects of Fh15 on macrophage phenotype and function. As hallmark cells of both the innate and adaptive immune responses, macrophages perform vital functions in pathogen clearance and in the initiation or resolution of inflammation.

## RESULTS AND DISCUSSION

### LPS-challenged mice treated with Fh15 exhibited a lower mortality rate than their untreated counterpart

In animal models, the dose of LPS is an important factor to consider, as well as the genetic background of the animals that will be used. In humans, concentrations as low as 2–4 ng/kg body weight (BW) are sufficient to induce a cytokine storm (9), whereas mice are less susceptible and require higher, lethal doses (LD50) of LPS ranging from 10 to 25 mg/kg BW to induce septic shock (10). Among commonly used strains, BALB/c mice are highly susceptible to the inflammatory effects of LPS exposure compared to C57BL/6 mice (4, 11). This increased susceptibility is primarily attributed to their tendency to mount a T helper-2 type response (12), which is associated with relatively impaired bactericidal activity (4) and a more pronounced inflammatory response in multiple tissues (11). These characteristics make the BALB/c mouse strain an excellent model for evaluating new therapeutic agents for bacterial septic shock, as untreated animals rapidly succumb to the disease (13).

In a previous study aimed to explore the therapeutic potential of Fh15, we demonstrated that a single i.p. injection of Fh15 (50 μg), administered to female BALB/c mice 1 h after LPS injection (7 mg/kg BW), significantly suppressed the cytokine storm. This effect was confirmed at 12 h following the LPS challenge (8). In the present study, we evaluated the capacity of different Fh15 concentrations (50, 100, and 200 μg) to prevent mortality in a model of endotoxic shock induced by a lethal dose of 12 mg/kg BW of LPS. As in our previous study, Fh15 treatment was administered 1 h after the LPS challenge, corresponding to the time frame in which pro-inflammatory cytokines, such as TNF-α, IL-6, and IL-1β, reach peak serum concentrations following i.p. injection of LPS (14).

At the humane endpoint of 18 h following the LPS challenge, all mice injected with LPS and treated with placebo (PBS) exhibited severe signs of endotoxic shock, including reduced physical activity, lethargy, ruffled fur, closed eyes, and altered respiration (Table S1). These symptoms worsened over time, and most animals either died or were euthanized before 24 h post-challenge. A previous study using male BALB/c mice (8 weeks old) challenged intraperitoneally with 10 mg/kg BW LPS reported a median survival rate of 66.7% (15). In contrast, our study employed a higher LPS dose that resulted in lethality for all animals (LD100). This may be attributed, in part, to sex-based differences, which are recognized as factors influencing the natural progression of sepsis in untreated animals (16).

**Fig 1:**
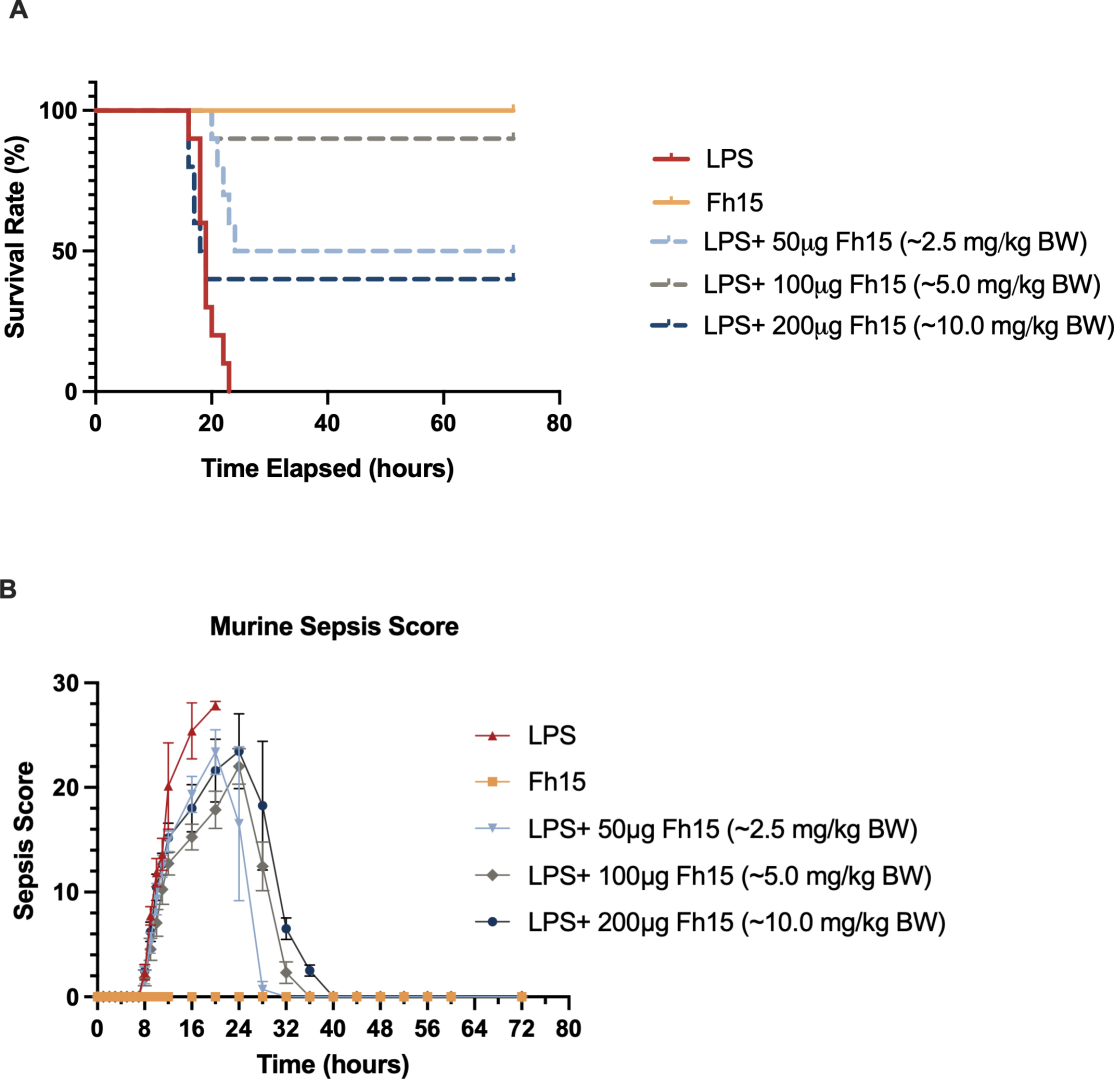
Fh15 protects mice from septic shock, promoting survival. Eight to ten weeks old BALB/c mice were injected i.p. with a lethal dose of LPS (12 mg /kg BW) and treated 1 h later with an i.p. injection of 50 µg (~2.5 mg/kg BW), 100 µg (~5.0 mg/kg BW), or 200 µg (~10 mg/kg BW) Fh15. Animals only injected with LPS (*n* = 10) were used as controls. (**A**) Animals were monitored for 72 h, and mortality was scored. (**B**) Murine sepsis score (MSS) over time in mice that were administered LPS alone or treated with Fh15. MSS was established based on appearance, level of consciousness, activity, response to stimuli, eyes, and respiration rate and quality. Each of these variables is given a score between 0 and 4, with higher scores indicating more severe clinical impairment. Mice with MSS at a given time point greater than 25 or with points ascribed to respiratory rate or quality increased by more than 3 were sacrificed. BW, body weight. ***P = 0.0002, **P = 0.0025; ns, no significant differences. Survival was analyzed by Kaplan–Meier survival curves. Statistical significance between groups was determined using the log-rank (Mantel–Cox) test with Holm–Šídák correction for multiple comparisons using GraphPad Prism version 10.

Remarkably, mice treated with Fh15, regardless of dose, exhibited higher survival rates ranging from 40% to 90%. Specifically, mice treated with 50 μg Fh15 showed a median survival rate of 50%, which was significantly higher than that of the LPS control group (*P* = 0.0025). Deaths in this group occurred between 14 and 22 h following the LPS challenge. Notably, signs of endotoxemia progressively resolve in surviving animals, and by the humane endpoint of 72 h, all mice treated with 50 μg Fh15 had returned to normal activity levels (Fig. 1; Table S1).

The group treated with 100 μg Fh15 exhibited the highest median survival rate (90%) (*P* = 0.0002). Only one animal in this group died approximately 20 h following the LPS challenge, while all the remaining mice displayed minimal clinical signs of endotoxemia and appeared healthy at 72 h. Interestingly, the lowest median survival rate (40%) was observed in mice treated with 200 μg Fh15. As observed in the other treatment groups, animals that succumbed did so within 24 h following the challenge, whereas survivors exhibited milder symptoms and fully recovered by 72 h. No statistically significant difference in survival was observed between the LPS-only group and the group treated with 200 μg Fh15. Survival data represent the average of two independent experiments conducted on separate days, which yielded comparable results (Fig. 1).

Considering that the average body weight of an approximately 8-weeks-old female BALB/c mouse ranges from 20 to 25 g, the administered concentrations of Fh15 (50, 100, or 200 μg) correspond to approximately 2.5, 5.0, and 10 mg/kg BW, respectively. Among these, the 5.0 mg/kg BW (100 μg) was identified as the optimal dose, as it maximized survival in this model.

**Fig 2:**
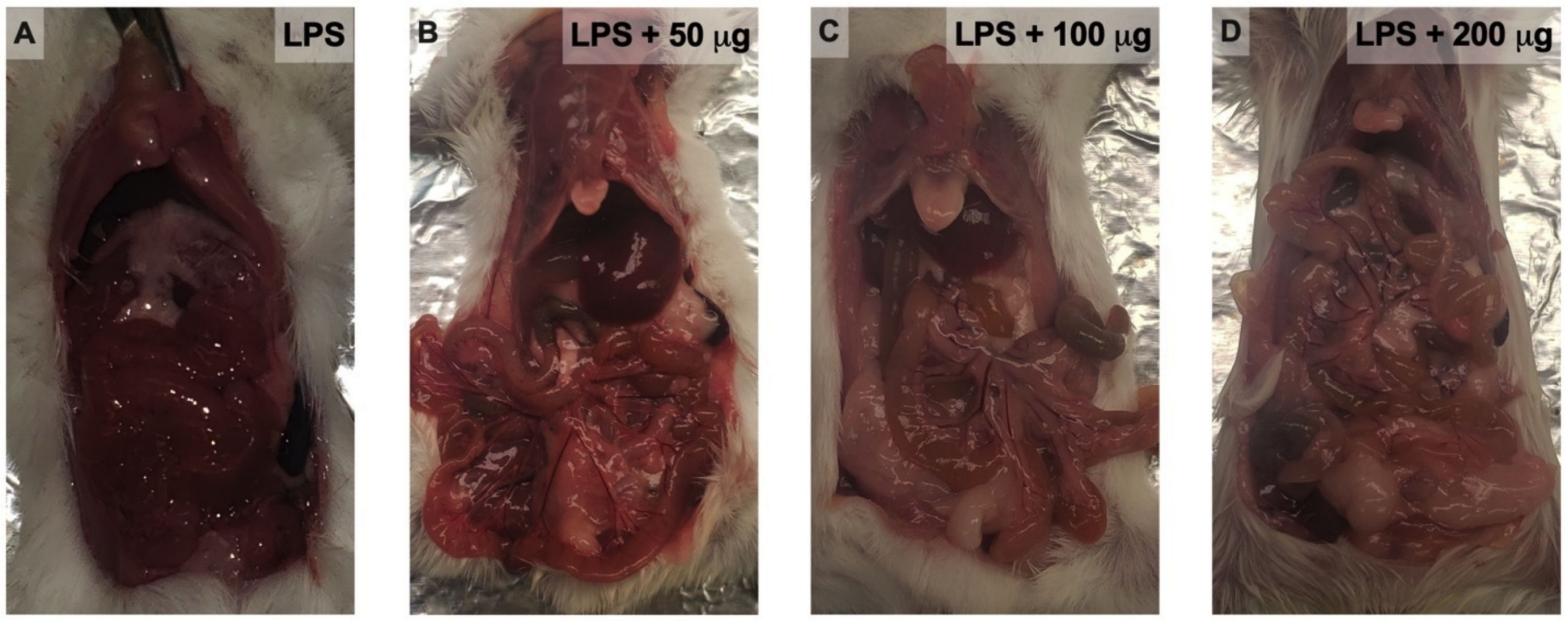
Macroscopic examination of the peritoneal cavity after LPS challenge. BALB/c mice were injected with a lethal i.p. injection of LPS (12 mg/kg BW) and 1 h later were treated with an i.p. injection of 50, 100, or 200 μg Fh15. Animals that were treated with a placebo (PBS) were used as controls. Pictures show that the peritoneal cavity of Fh15-treated animals (**B, C, D**) maintained a healthy gross appearance compared to the inflamed and bloody appearance observed in LPS-control mice (**A**).

Consistent with mortality results, gross examination of the abdominal cavity of animals that died during monitoring, particularly those in the LPS-control group, showed clear evidence of hemorrhage. In contrast, animals treated with Fh15 that survived the challenge, specifically those that were treated with 100 μg Fh15, displayed a gross macroscopic appearance comparable to that of mice injected with PBS or Fh15 alone (Fig. 2). Unfortunately, histopathological analyses of major organs such as the lungs, liver, and kidneys, which are the most affected during septic shock, were not performed. Additionally, the effects of Fh15 on neutrophils and other immune cells at the tissue level were also not evaluated. These studies, together with pharmacodynamic and pharmacokinetic analyses, will be essential to more accurately determine the safety profile and tissue-level effects of this therapeutic approach and will be the focus of future investigations.

### Fh15 treatment suppresses the cytokine storm over time

Given that individuals who can effectively control the inflammatory response during endotoxemia are more likely to survive (17), we analyze the levels of pro-inflammatory cytokines in serum samples from mice treated with the different doses of Fh15 following LPS challenge. We initially focused on three inflammatory cytokines: TNF-α, IFN-γ, both considered biomarkers of sepsis, and IL-12p70, a strong inducer of IFN-γ production during sepsis (17, 18). In addition, we measured levels of IL-10, a potent regulatory cytokine that plays a dual role during sepsis by either suppressing inflammation or exacerbating immunosuppression (19).

**Fig 3:**
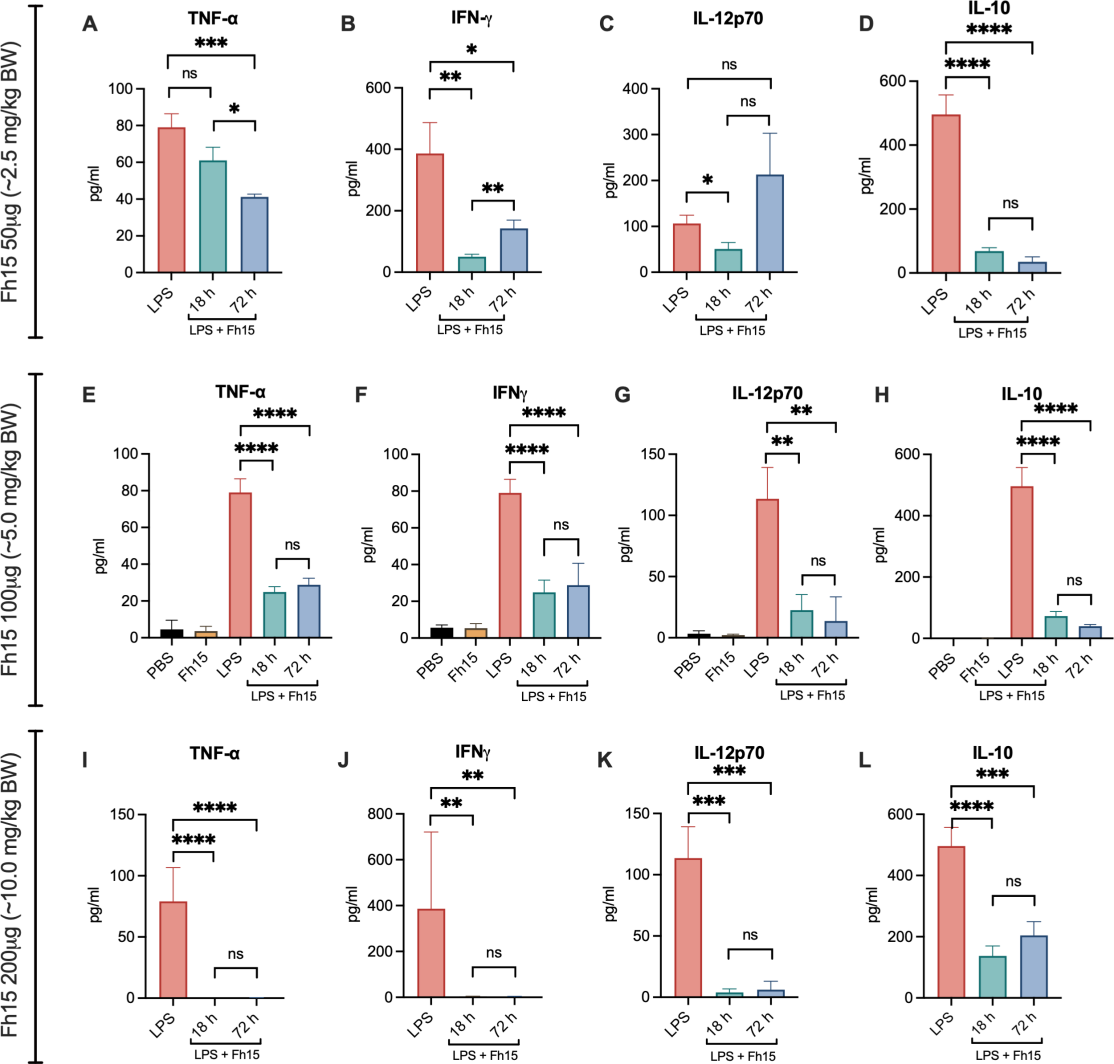
Fh15 suppresses the induction of pro-inflammatory cytokines *in vivo* elicited by LPS. Eight to ten weeks old BALB/c mice were injected i.p. with 12 mg/kg BW and 1 h later animals were treated with a single i.p. dose of 50 µg (~2.5 mg/kg BW), 100 µg (~5.0 mg/kg BW), or 200 µg (~10 mg/kg BW) and euthanized 18 h after LPS-challenging. Animals treated with Fh15, which survived for 72 h after the challenge infection, were bled at 72 h (end of experiment). Animals that only received the i.p. injection with LPS (*n* = 5) were used as a positive control. These animals died between 12 and 24 h and were bled shortly before death. Animals only injected with Fh15 (*n* = 5) or PBS (*n* = 5) were used as negative controls. (**A–D**) Represents serum cytokine levels in animals treated with 50 μg of Fh15 following LPS challenge, showing a significant reduction in TNF-α (***P = 0.002), IFN-γ (*P = 0.0392, **P = 0.0077), IL-12p70 (*P = 0.0203), and IL-10 (****P < 0.001) compared to untreated animals. (**E–H**) Also, serum cytokine levels in animals treated with 100 μg of Fh15 following LPS challenge demonstrate a significant suppression of TNF-α (****P < 0.0001), IFN-γ (****P < 0.0001), IL-12p70 (**P = 0.0014, **P = 0.0028), and IL-10 (****P < 0.0001) compared to untreated animals. (**I–L**) Finally, following LPS challenge, animals administered with 200 μg of Fh15 exhibit a significant reduction in serum levels TNF-α (****P < 0.0001), IFN-γ (**P = 0.0035, **P = 0.0036), IL-12p70 (***P = 0007, ***P = 0.006), and IL-10 (***P = 0.006, ****P < 0.0001), compared to animals that did not receive the treatment. Statistical significance was determined by one-way ANOVA, followed by Tukey’s multiple-comparisons test using GraphPad Prism version 10. Data are shown as mean ± SEM. ns, no significant differences.

Particularly, all animals in the LPS-control group exhibited markedly elevated serum levels of TNF-α, IFN-γ, IL-12p70, and IL-10 (Fig. 3), ranging from 43.98 to 137.99 pg/mL (average 79.045 ± 26.667 pg/mL), 35.44 to 1,008.86 pg/mL (average 406.043 ± 361.079 pg/mL), 29.30 to 371.22 pg/mL (average 120.401 ± 101.692 pg/mL), and 166.85 to 1,128.29 pg/mL (average 496.245 ± 265.764 pg/mL), respectively (Table 1). All these cytokine concentrations were significantly higher than those detected in sera from LPS-challenged animals treated with Fh15 or from PBS and Fh15 control groups (*P* < 0.0001). Since all animals in the LPS-control group died within 24 h of challenge, cytokine determinations were performed on serum samples collected from animals showing severe signs of endotoxemia that were about to die and humanely euthanized.

**TABLE 1:** Effect of different doses of Fh15 on concentrations of TNFα, IFNγ, IL-12p70, and IL-10 in serum from female BALB/c mice exposed to 12 mg/kg body weight LPS, a dose that resulted lethal for all animals (LD100) in less than 24 h^*a*^^,^^*b*^^,^^*c*^

LPS (12 mg/kg)						
	18 h			72 h		
	Concentration Range (ng/mL)	Average ± SD (ng/mL)	Reduction %	Concentration Range (g/mL)	Average ± SD (ng/mL)	Reduction %
TNFα	43.98 to 137.99	79.045 ± 26.667	0	ND^*d*^	ND	ND
IFNγ	35.44 to 1,008.86	406.043 ± 361.079	0	ND	ND	ND
IL-12p70	29.3 to 371.22	120.401 ± 101.692	0	ND	ND	ND
IL-10	166.85 to 1,128.2	496.245 ± 265.764	0	ND	ND	ND
LPS (12 mg/kg) + Fh15 (50 µg = 2.5 mg/kg)						
TNFα	24.71 to 122.88	61.082 ± 25.753	22.72	35.71 to 42.25	39.894 ± 2.448	49.53
IFNγ	28.32 to 98.38	50.846 ± 23.175	87.48	29.04 to 250.09	142.894 ± 75.750	64.81
IL-12p70	10.9 to 146.64	67.985 ± 56.073	43.53	0 to 243.52	69.810 ± 91.117	42.01
IL-10	0 to 117.34	68.832 ± 36.074	86.12	20.11 to 60.55	35.361 ± 14.022	92.87
LPS (12 mg/kg) + Fh15 (100 µg = 5 mg/kg)						
TNFα	14.79 to 31.59	28.750 ± 11.404	63.62	7.75 to 45.64	24.842 ± 5.956	68.57
IFNγ	12.83 to 34.11	20.128 ± 8.285	95.04	4.83 to 28.63	19.639 ± 7.173	95.16
IL-12p70	10.90 to 46.31	31.675 ± 14.635	73.69	0 to 50.91	13.720 ± 18.421	88.60
IL-10	8.79 to 204.02	72.907 ± 50.810	85.31	19.59 to 73.47	39.779 ± 17.415	91.98
LPS (12 mg/kg) + Fh15 (200 µg = 10 mg/kg)						
TNFα	0	0	100.0	0 to 1.17	0.117 ± 0.351	99.85
IFNγ	2.62 to 5.67	5.040 ± 0.630	98.76	0 to 6.22	2.402 ± 1.961	99.41
IL-12p70	0.95 to 8.71	3.939 ± 2.662	96.73	0 to 13.35	6.215 ± 6.252	94.83
IL-10	38.47 to 258.53	137.424 ± 85.632	72.31	50.85 to 429.29	204.024 ± 143.443	58.88

^*a*^ Reduction % = 1 − [C2/C1] × 100.

^*b*^ Where C1 = average cytokine concentration in the LPS group at 24 h.

^*c*^ C2 = average cytokine concentration of each LPS + Fh15 treated group at 18 and 72h.

^*d*^ ND, not determined.

Notably, healthy animals that received Fh15 alone exhibited very low or undetectable levels of TNF-α, IFN-γ, IL-12p70, and IL-10, which remained consistently low for 72 h, indicating that Fh15 does not induce inflammatory responses. These findings are consistent with previous reports from our research group using C57BL/6 and BALB/c mice, as well as rhesus macaques, suggesting that Fh15 is well-tolerated (6, 7, 20). Although Fh15 alone did not elicit pro-inflammatory cytokines, it significantly reduced cytokine levels following LPS challenge (Fig. 3).

At 18 h post-challenge, mice treated with 50 μg Fh15 (~2.5 mg/kg) exhibited an average TNF-α concentration of 61.082 ± 25.753 pg/mL, representing a 22.72% reduction, compared to the LPS-control group. By the 72 h endpoint, TNF-α levels were further reduced to 39.894 ± 2.448 pg/mL (*P* = 0.0164), corresponding to a 49.53% reduction. At the humane endpoint of 18 h, mice treated with 100 μg Fh15 (~5 mg/kg) displayed a 63.62% reduction in TNF-α levels compared to the LPS-control group (average 28.750 ± 11.404 pg/mL; *P* < 0.0001), representing a 2.80-fold higher reduction compared to the 50 μg dose (2.5 mg/kg). At the humane endpoint of 72 h, TNF-α suppression increased slightly to 68.57% (average 24.842 ± 5.956 pg/mL), corresponding to 1.38-fold higher compared to the dose of 50 μg (~2.5 mg/kg).

Unexpectedly, the highest dose tested (200 μg Fh15; ~10 mg/kg) resulted in near-complete (~100%) suppression of TNF-α levels, which was sustained through the 72 h following the LPS challenge. Paradoxically, this group exhibited the highest mortality rate (40%). Similar trends, with slight fluctuations, were observed for IFN-γ and IL-12p70. While the 50 μg dose (2.5 mg/mL BW) seems to increase IFN-γ and IL-12p70 levels by 72 h, the 100 μg dose (~5 mg/kg) maintains moderate-to-high suppression of all cytokines over time. In contrast, the 200 μg dose (~10 mg/kg) produces sustained suppression of all measured cytokines over time (Fig. 3; Table 1).

The inflammatory response induced by LPS is known to activate NF-κB, a critical transcription factor that regulates the expression of genes encoding inflammatory cytokines and other immune mediators essential for host defense (21). The pronounced suppression of TNF-α, IFN-γ, and IL-12p70 observed with the 200 μg dose (~10 mg/kg) of Fh15 suggests near-complete suppression of NF-κB signaling pathway. This finding indicates that administering Fh15 at such a high dose could be detrimental rather than beneficial for the host, as NF-κB signaling pathway is also essential for immune homeostasis and tissue protection (22). Importantly, the cytokine suppression observed in female BALB/c mice treated with Fh15 was similar to that previously reported in female C57BL/6 mice treated for 18 h with Fh12, the native variant of *F. hepatica* FABP (20). Given that C57BL/6 mice tend toward Th1-immune responses and are less susceptible to endotoxemia than BALB/c (12), and considering that Fh12 and Fh15 share similar immunological and anti-inflammatory properties (8), it is reasonable to speculate that Fh15 may also improve survival rate and reduce cytokine storm severity in the C57BL/6 strain.

Another key finding of this study was the elevated IL-10 levels observed in mice subjected to LPS challenge alone. Although IL-10 is considered a regulatory cytokine with anti-inflammatory functions, it can act as a double-edged sword during sepsis. Studies have demonstrated that early in sepsis, IL-10 is secreted in large amounts to dampen inflammation; however, sustained elevated levels during later stages correlate positively with increased mortality due to immunosuppression and impaired bacterial clearance (23). In our study, IL-10 levels induced by LPS were reduced by 86.12% at the endpoint of 18 h and 92.87% at the endpoint of 72 h in mice treated with 50 μg Fh15 (~2.5 mg/kg), and by 85.31% and 91.99% at the endpoint of 18 and 72 h, respectively, in mice treated with 100 μg Fh15 (~5 mg/kg). These reductions are consistent with the improved survival rate observed in these groups. Moreover, treatment with 200 μg Fh15 (~10 mg/kg) reduced IL-10 levels by only 72.31% at 18 h and or 58.88% at 72 h (Table 1), suggesting that this dose may fail to prevent the immunosuppressive state associated with the excessive IL-10, thereby contributing to increased mortality. However, since we do not have available basic safety data (e.g., body weight trajectories and histopathological analysis), excessive immunosuppression or off-target toxicity associated with high Fh15 doses cannot be ruled out. Collectively, these results demonstrate that sustained reduction of inflammatory cytokines observed at 18 and 72 h post-challenge indicates that the 100 μg dose (~5 mg/kg) optimally suppresses the cytokine storm, increases the survival rate, and decreases the risk of immunosuppression.

### Fh15 treatment suppresses serum levels of IL-1β, IL-6, and C-reactive protein but increases IL-37 levels

Having demonstrated that the 100 μg dose of Fh15 maximized both survival rate and the reduction of pro-inflammatory cytokines, we proceeded to measure serum levels of C-reactive protein (CRP), IL-37, IL-1β, and IL-6 in animals that were treated with this dose. These results were compared with those obtained from sera of the LPS-control group. CRP is an inflammatory marker that, although not specific for sepsis, is elevated during septic shock and can serve as an indicator of inflammation and disease progression (24). Both IL-1β and IL-6 are pro-inflammatory cytokines secreted primarily by macrophages in response to inflammatory stimuli and are key mediators of the systemic inflammatory response during sepsis. Elevated IL-6 levels are a potent marker of early sepsis and strongly correlate with disease severity (25). Similarly, excessive and prolonged IL-1β production contributes to organ injury, neuroinflammation, and other severe complications associated with sepsis (26). Conversely, IL-37, a member of the IL-1 family, known as a suppressor of inflammation (27), plays a critical role in inhibiting the excessive immune responses during sepsis (28).

**Fig 4:**
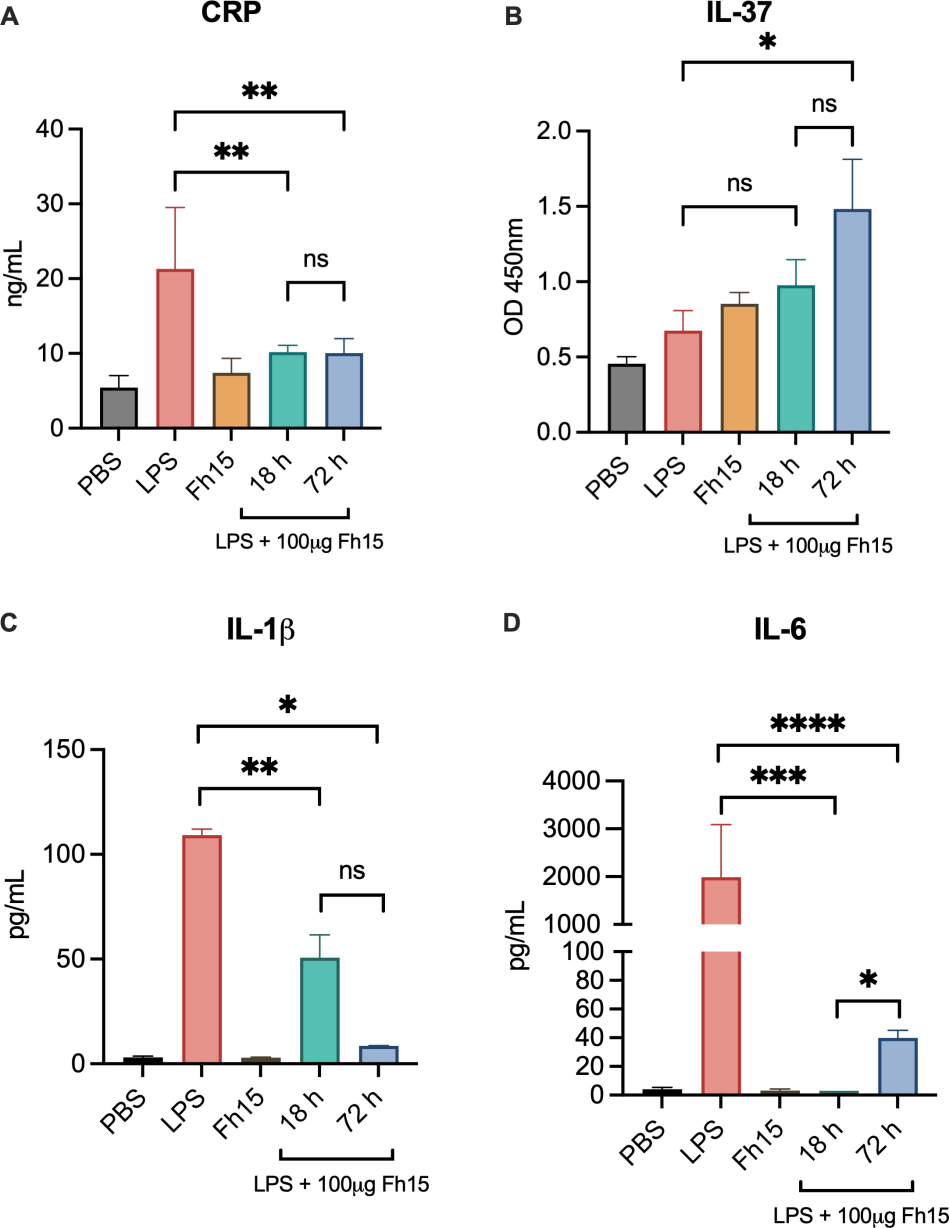
Fh15 suppresses levels of serum CRP, IL-1β, and IL-6, whereas it increases levels of IL-37. (**A**) Represents the levels of serum C-reactive protein (**P = 0.0021, **P = 0.0019), (**B**) IL-37 (*P = 0.0289), (**C**) IL-1β (*P < 0.0001, **P = 0.0001), and (**D**) IL-6 (*P > 0.9999, ***P = 0.0039, ****P = 0.0150). Statistical significance between groups was determined by one-way ANOVA, followed by Tukey’s multiple-comparisons test using GraphPad Prism version 10. For all tests, a *P* value of <0.05 was considered significant. Data are shown as mean ± SEM. ns, no significant differences.

As shown in our results, animals injected with Fh15 alone exhibited serum CRP concentrations ranging from 5.02 to 9.74 ng/mL (average 7.407 ± 1.743 ng/mL), which were not significantly different from background levels detected in the PBS-control group (average 5.421 ± 1.444 ng/mL). In contrast, animals in the LPS-control group displayed elevated CRP levels ranging from 15.05 to 33.23 ng/mL (average 21.277 ± 7.34 ng/mL), indicating a pronounced inflammation (Fig. 4). Treatment with Fh15 reduced CRP levels by 2.09-fold at the endpoint of 18 h (average 10.169 ± 0.819 ng/mL), and this reduction was sustained for 72 h after challenge (average 10.043 ± 1.717). This finding was consistent with the significant suppression of IL-1β and IL-6. At 18 h post-challenge, mice treated with 100 μg Fh15 (~5.0 mg/kg) exhibited average serum concentrations of 50.720 ± 28.592 pg/mL for IL-1β and 2.995 ± 1.597 pg/mL for IL-6, corresponding to reductions of 53.57% and 99.85%, respectively, compared to the LPS-control group. At the endpoint of 72 h, the reduction percentages were 92.16% for IL-1β (8.560 ± 0.173 pg/mL) and 97.989% for IL-6 (39.95 ± 9.042) when compared to the LPS-control group. The observation that both IL-1β and IL-6 were significantly decreased in Fh15-treated mice and that these reductions were maintained over time reinforces the strong anti-inflammatory capacity of this recombinant molecule.

Importantly, IL-37 was very low in the LPS-control (average OD at 450 nm 0.602 ± 0.251), PBS-control (average 0.455 ± 0.092 OD at 450 nm), and Fh15 groups (average 0.852 ± 0.151 OD at 450 nm). In contrast, IL-37 levels increased by 2.107-fold in septic mice treated with Fh15 (average 1.269 ± 0.665 OD at 450 nm). These results suggest that Fh15 may promote increased IL-37 levels as part of its mechanism for suppressing the cytokine storm and other inflammatory markers such as CRP.

### Fh15 suppresses classical macrophage activation, impairing phagocytic function and ability to interact with T-cells

Because sepsis is characterized by an imbalance in the immune response driven by excessive M1-type macrophage polarization, which contributes to cytokine storm and organ dysfunction (29), we aimed to determine whether Fh15 could exert any influence on the macrophage polarization. To this end, bone marrow-derived macrophages (BMDMs) from naïve female BALB/c mice were isolated, differentiated, and stimulated overnight at 37°C and 5% CO_2_ with previously optimized concentrations of LPS (1 ng/mL), Fh15 (10 μg/mL) or a combination of LPS (1 ng/mL) + Fh15 (10 μg/mL) (6, 8).

The mRNA expression of TNF-α, IL-10, nitric oxide synthase-2 (NOS2), cluster differentiation 206 (CD206), and resistin-like molecule alpha-1 (Fizz1) was quantified by real-time RT-PCR (RT-qPCR). In parallel, flow cytometry was used to assess surface expression of CD80 and CD38, as well as intracellular NOS2 protein levels. Additionally, based on our previous findings demonstrating that Fh12 suppresses CD14-coreceptor expression to prevent LPS-induced TLR4 activation (20), in the present study, we examined whether Fh15 exerts a similar effect.

**Fig 5:**
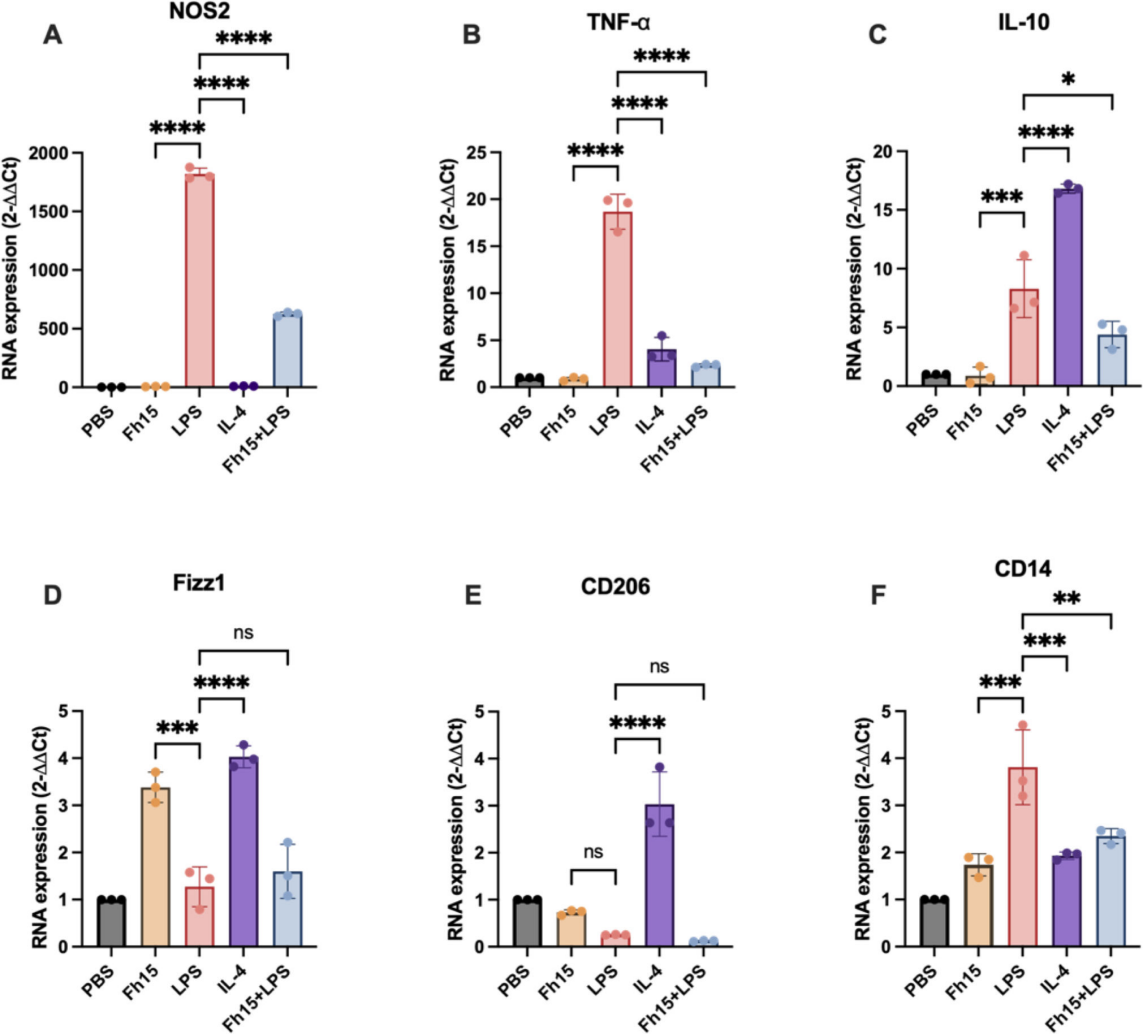
Fh15 increases IL-10 expression, whereas reduces the TNF-α and NOS2 expression. Bone marrow-derived macrophages were cultured in a 24-well plate (1.0 × 10^6^ cells/well) and treated with LPS (100 ng/mL), IL-4 (20 ng/mL), Fh15 (10 µg/mL), or Fh15 + LPS. Cells were detached for RNA extraction and subjected to amplification by RT-qPCR using specific primers for NOS2, TNF-α, IL-10, Fizz1, and CD206. Cells stimulated with LPS showed high expression levels of NOS2, TNF-α, IL-10, and CD14 indicating macrophages are classically activated (M1). Despite the treatment with Fh15 alone showed minimal expression of these markers, it was able to significantly suppress the LPS-induced levels of (**A**) NOS2 (****P < 0.0001), (**B**) TNF-α (****P < 0.0001), and (**F**) CD14 (**P = 0.0056) while increase (**C**) IL-10 (*P < 0.0236). (**D**) Compared to LPS, Fh15 increased the expression of Fizz1 (*P* = 0.0002) and failed to increase the expression of CD206 (**E**). Moreover, Fh15 also failed to increase these markers in the presence of LPS, suggesting that Fh15 is unable to promote M2-type polarization. Statistically significant differences were determined by using one-way ANOVA with Tukey's multiple comparisons test using GraphPad Prism version 10. Data are shown as mean ± SD. ns, no significant differences.

**Fig 6:**
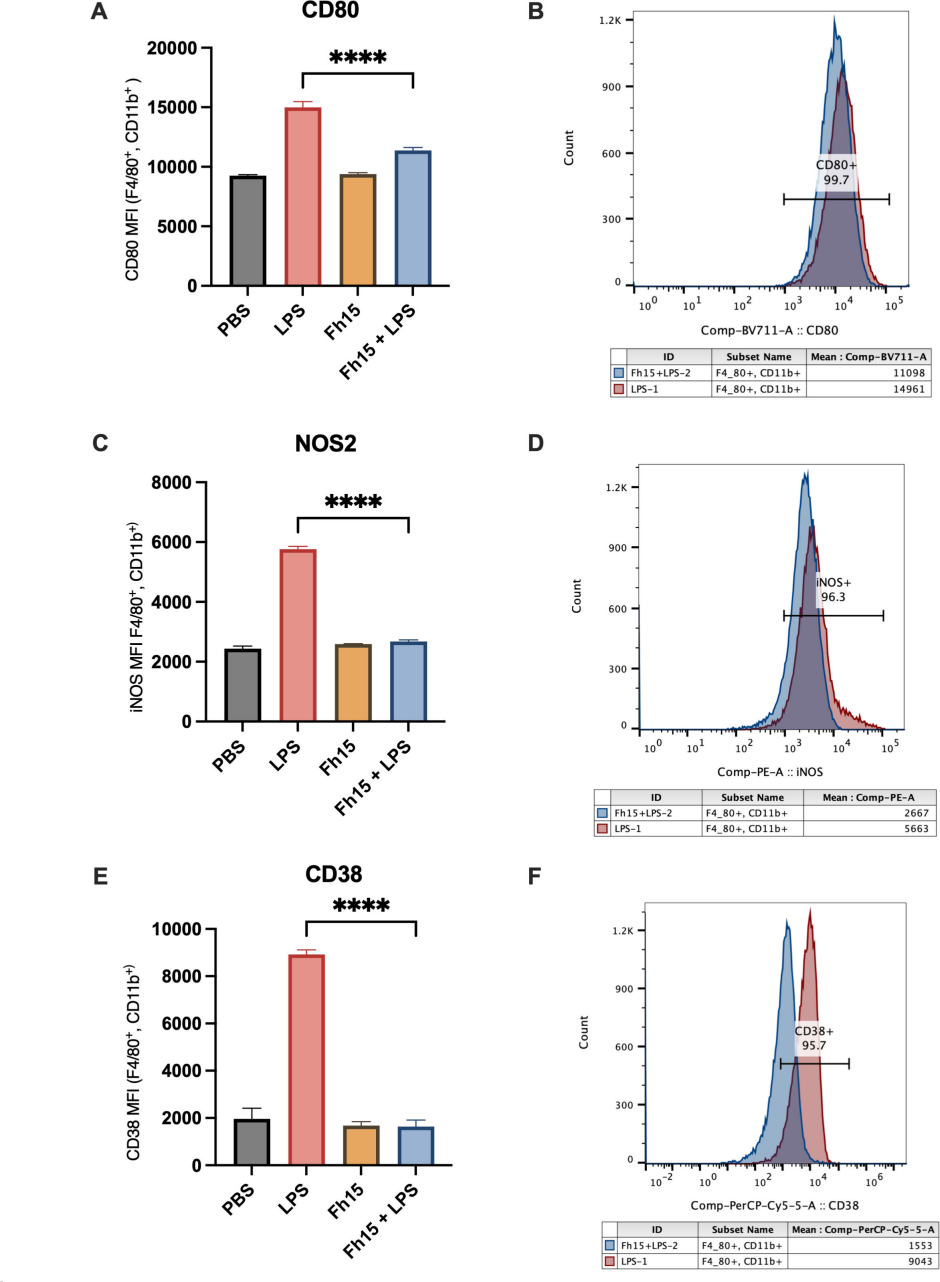
Fh15 suppresses the expression of proteins associated with several inflammatory pathways within bone marrow-derived macrophages. Fh15 treatment significantly suppresses the expression of CD80, NOS, and CD38. (**A, C, E**) The bar graph displayed the mean fluorescent intensity (MFI). (**B, D, F**) The overlaid histograms illustrate the expression in the F4/80+CD11b+ cell population, comparing samples from the LPS group (red) and the Fh15+LPS group (blue). Flow cytometry data were analyzed using FlowJo software (version 10.6.2), and statistically significant differences were determined by using one-way ANOVA with Tukey’s multiple comparisons test using GraphPad Prism version 10. ****P < 0.0001. Data are shown as mean ± SD.

As our results demonstrate, macrophages stimulated with LPS expressed high levels of CD14, NOS2, TNF-α, CD38, and IL-10 (Fig. 5 and 6). CD14 is a key co-receptor for TLR4, enhancing its ability to recognize LPS and subsequently triggering an immune response that leads to inflammation (30). NOS2 is a crucial enzyme overexpressed in M1-type macrophages, which play an essential role during the inflammatory response by producing nitric oxide (NO). While NO can be beneficial in fighting infections, excessive production may contribute to tissue damage and increased pathology (31). M1-type macrophages are also characterized by secreting large amounts of TNF-α, a key inflammatory cytokine overproduced in response to LPS, leading to the cytokine storm (32). Moreover, M1-type macrophages overexpress high levels of CD38, another M1-type marker associated with a pro-inflammatory cytokine profile (33), which plays essential roles in the (cADPR) synthesis, regulating the intracellular calcium through TLR4 (34).

In contrast, macrophages treated with Fh15 alone exhibited minimal expression of these markers. Importantly, Fh15 significantly reduced LPS-induced expression of NOS2 (*P* < 0.0001), TNF-α (*P* < 0.0001), CD14 (*P* = 0.0056) (Fig. 5), and CD38 (P<0.0001) (Fig. 6). These results indicate that Fh15 suppresses M1-type macrophage polarization, consistent with our recent proteomics study with murine macrophages in which we demonstrated that Fh15 modulates inflammatory responses by modulating the NF-κB and NOS2 pathways (35).

Moreover, the IL-10 overexpression observed in BMDMs stimulated with LPS alone is consistent with our *in vivo* results and reinforces the evidence that LPS, through its interaction with TLR4, activates macrophages to produce both inflammatory and anti-inflammatory cytokines. Normally, M2-type macrophages overexpress CD206 and Fizz1, as observed following IL-4 stimulation, a Th2 cytokine that is a strong M2-type inducer. Although we observed that Fh15 did not induce CD206 expression, it did overexpress Fizz1 at levels comparable to IL-4 stimulation (Fig. 5). However, in contrast to expectations, macrophages treated with Fh15 and subsequently stimulated with LPS expressed low levels of both CD206 and Fizz1, suggesting that Fh15 may not directly promote M2 polarization. Instead, the simultaneous suppression of M1-associated markers and the limited expression of M2-associated genes suggest that Fh15 drives macrophages toward a “deactivated” or “regulatory” phenotype. Such a phenotype is characterized by reduced responsiveness to inflammatory stimuli and the promotion of tissue protection and homeostasis, rather than a direct anti-inflammatory polarization. This interpretation aligns with the broader immunomodulatory profile of helminth-derived molecules, which often reprogram macrophages toward a regulatory phenotype that mitigates inflammation without fully suppressing immune competence (36).

The observed reduction in TNF-α expression induced by Fh15 treatment in LPS-stimulated macrophages is also consistent with our previous TMT-based proteomics findings, in which BMDMs treated with LPS + Fh15 showed a significant downregulation of the relative abundance of TNF-α protein compared to cells only stimulated with LPS alone. This dysregulation was subsequently validated by either western blot or ELISA quantification of TNF-α secretion in cell culture supernatants (35). Although in the present study, IL-10 and TNF-α concentrations were not measured in the cell culture supernatants, the consistent downregulation of proinflammatory gene expression suggests a broader modulatory effect of Fh15 on cytokine signaling pathways.

**Fig 7:**
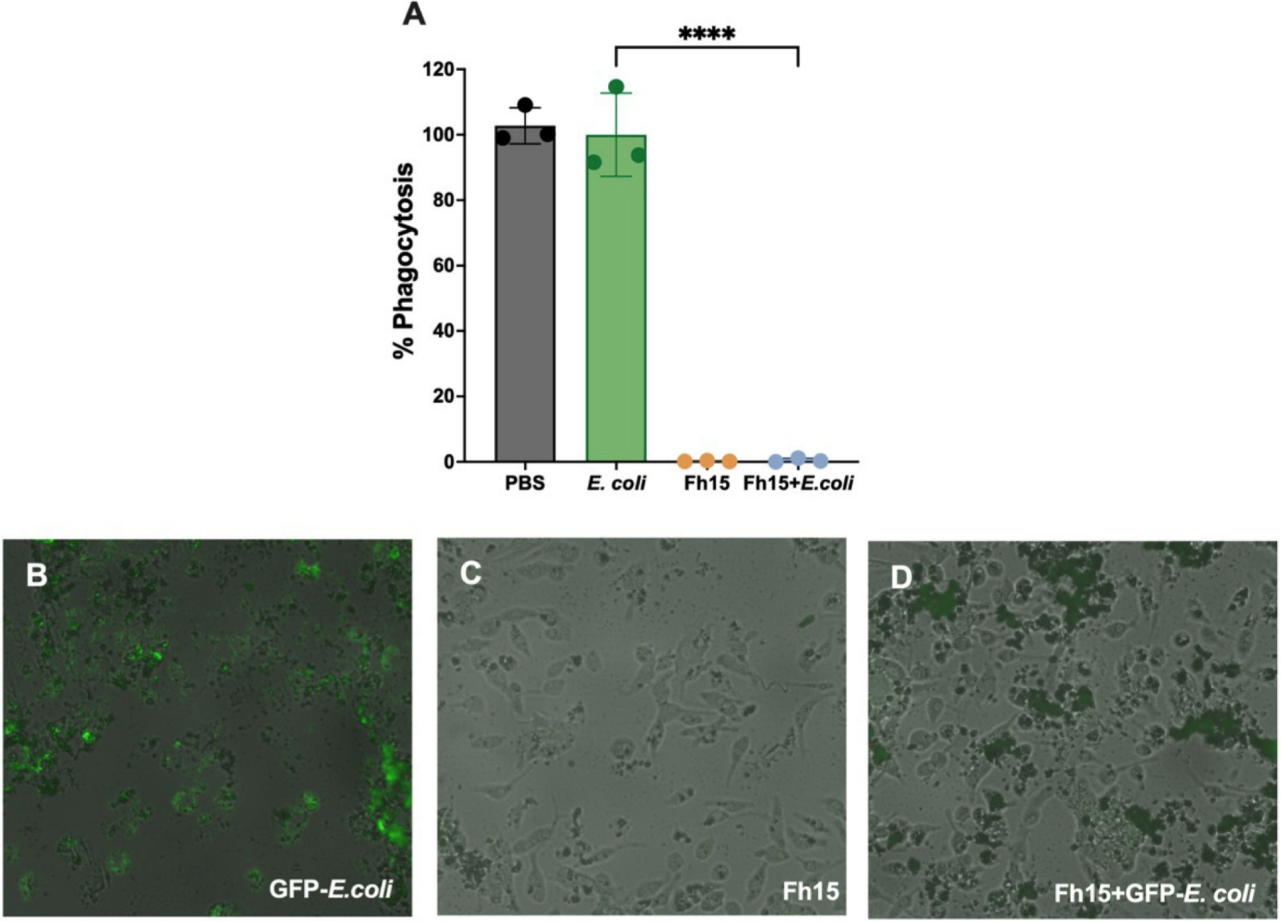
Fh15 limits the bacterial phagocytosis of bone marrow-derived macrophages. Cells were seeded overnight at 1.0 × 10^5^ of viable cells/well in a 96-well plate. The next day, the cells were treated with 10 µg of Fh15 for 1 h at 37°C prior to the addition of 5 µL of heat-killed, GFP-labeled *E. coli* particles. Phagocytosis was conducted for 6 h, and the amount of engulfed *E. coli* was determined by scanning all experimental and control wells in the plate reader at Ex/Em at 490/520 nm and using fluorescent microscopy. (**A**) Fh15 suppressed the phagocytic activity of macrophages by 100% (****P < 0.0001). Statistically significant differences were determined by using one-way ANOVA with Tukey’s multiple comparisons test using GraphPad Prism version 10. Data are shown as mean ± SD. (**B–D**) Overlay of Bright Field and GFP fluorescence images captured using NIS-Elements software, illustrating cellular morphology alongside GFP expression.

Another important finding of this study is that Fh15 significantly suppressed the phagocytosis of *E. coli* by macrophages (*P* < 0.0001) (Fig. 7). Phagocytosis is a fundamental macrophage function that acts as sentinels that detect, engulf, and destroy pathogens during the early phase of the immune response. This function is particularly prominent in M1-type macrophages and contributes to inflammation by releasing damage-associated molecular patterns that activate inflammasome and NF-κB signaling pathways (37). During sterile endotoxemia, suppression of phagocytosis may be beneficial by limiting immune over-activation and tissue damage in the absence of live bacteria. However, this is a complex issue, with potential disadvantages, and the loss of phagocytic activity could theoretically impair bacterial clearance. Nonetheless, in a previous study using a nonhuman primate model of sepsis, it was observed that prophylactic administration of Fh15 significantly decreased bacteremia (7), suggesting that its immunomodulatory effects can occur without compromising the microbial control. These findings suggest that Fh15 may fine-tune macrophage function rather than completely suppress it. Therefore, the suppression of macrophage phagocytic activity induced by Fh15 is consistent with the suppressive effect on M1-associated markers and the capacity of macrophages to release inflammatory mediators. Although M2-type macrophages also possess phagocytic capacity, this type of macrophages is involved in tissue repair and resolution of inflammation (37). Therefore, additional studies using polymicrobial sepsis models, such as cecal ligation and puncture, will be needed to determine whether the observed reduced phagocytosis represents a therapeutic effect by mitigating inflammation or a risk during infection with live bacteria. Further studies will focus on elucidating these interactions, which are important for the safe and effective translation of Fh15 as an immunomodulatory therapy.

Macrophages also play a critical role in adaptive immune response through antigen presentation to T-cells via major histocompatibility complex class-I or class-II (38). Effective T-cell activation requires macrophages to express, on their surface, co-stimulatory molecules such as CD80 (B7-1), which along with CD86, provides signals essential for promoting T-cell activation and proliferation in the context of a Th1 response (39). Since Th1 cells promote M1 polarization in macrophages, the significant suppression of CD80 expression (*P* < 0.0001) by Fh15 suggests a mechanism by which Fh15 limits the interplay between Th1 cells and M1-type macrophages, an interaction that is excessively activated during sepsis and can lead to tissue damage and mortality (40). This suppression may indirectly induce a deactivated state contributing to immune regulation and resolution of inflammation.

It is noteworthy that neutrophils are one of the earliest responders during sepsis and play an important role in bacterial clearance through the release of neutrophil extracellular traps (NETs). Extensive neutrophil infiltration and NETs formation have been observed in endotoxin-induced sepsis models and are strongly linked to tissue damage and multiple-organ failure (41). Although in the present study, we did not evaluate the neutrophil infiltration in tissues of septic animals, previous work demonstrated that Fh15 exerts a strong immunomodulatory effect on neutrophil and macrophage infiltration in a mouse model of colitis (42), suggesting a potential role in the regulation of innate immune cell trafficking during sepsis. However, the complex dual role of these cells in immune protection and immune damage needs further specific studies to determine whether Fh15 increases infiltration and NETs formation. Understanding these mechanisms will be key to fully elucidating the therapeutic potential of Fh15 in controlling excessive inflammation while preserving essential immune functions.

In summary, this study reinforces the anti-inflammatory capacity of Fh15 as a potential biotherapy *in vitro* and *in vivo,* using a mouse model of septic shock. Several findings stand out from the results being reported here: (i) a single intraperitoneal injection with an optimal dose of 100 μg Fh15 (~5 mg/kg) administered 1 h after an LD100 LPS-challenge can significantly maximize both survival rate and suppression of the cytokine storm; (ii) these therapeutic effects are associated with significant reduction in CRP and a concurrent increase in IL-37 levels; (iii) the optimized 100 μg Fh15 dose (5 mg/kg) limits excessive IL-10 production, preventing the immunosuppressive status that potentially contributes to the high mortality rate; and (iv) the suppression of CD14, TNF-α, NOS2, CD38, and IL-12p70 induced by Fh15 exerts a profound inhibitory effect on macrophage maturation and function, including phagocytic activity and their capacity to activate Th1-cells. Thus, this study represents a significant contribution to the development of a potential recombinant protein therapy that blocks either TLR4 activation or downstream signaling pathways, leading to inhibition of inflammatory molecules implicated in sepsis pathology.

## MATERIALS AND METHODS

### Recombinant Fh15

The recombinant Fh15 used in the present study was expressed in *Bacillus subtilis* as a fusion protein with a 6His-tag at the amino terminus and purified in a single step by using a Ni+-agarose column. After removal of endotoxins, the protein concentration was adjusted to 2.29 mg/mL, as determined by the Bradford method. Levels of endotoxin assessed by a Chromogenic Limulus Amebocyte Lysate assay (Lonza, Walkersville, MD) revealed that Fh15 contained only traces at levels <0.4 EU/mg. The purity of Fh15 was >90% as determined by densitometric analysis of the Coomassie blue-stained SDS-PAGE gel under reducing conditions and confirmed by LC-MS as previously described (35).

### Mouse model of endotoxemia

**Fig 8:**
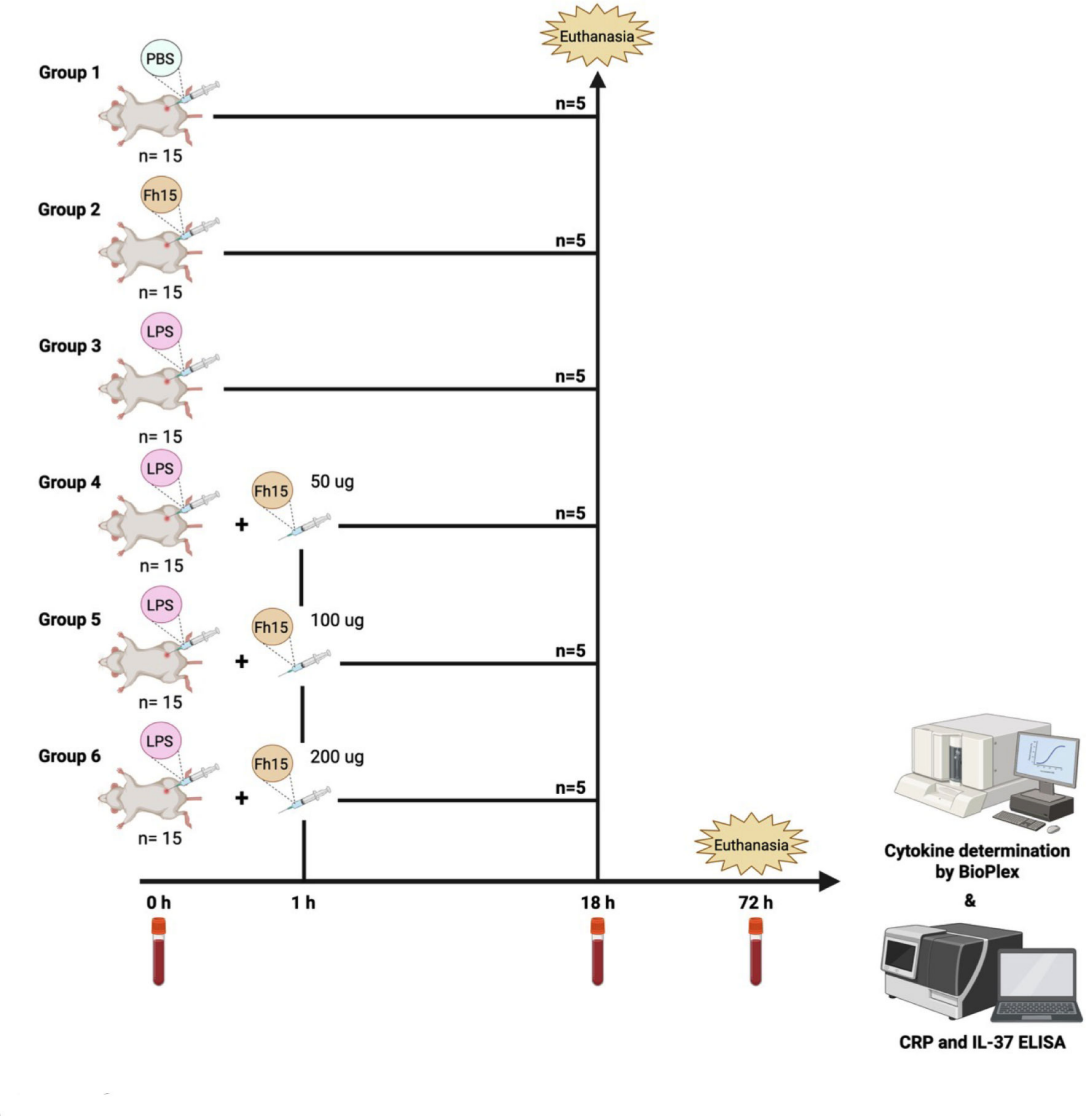
Experimental design for determining whether Fh15 can promote long-term pro-inflammatory cytokine reduction and prevent the mortality rate in a septic shock mice model. Female BALB/c (6–8 weeks old) mice were allotted into groups of 15 animals each. Groups 1 and 2 were the negative control groups, which will receive i.p. injection with PBS (placebo) or 100 μg Fh15, respectively. Group 3 is the sepsis control group and received i.p. with LPS (12 mg/kg BW) + equivalent PBS volume (placebo). Groups 4, 5, and 6 received first the LPS i.p. injection and 1 h later received a single i.p. injection with 50 µg (~2.5 mg/kg BW), 100 µg (~5.0 mg/kg BW), or 200 µg (~10 mg/kg BW) of Fh15, respectively. Five animals from each group were selected at random for bled out and euthanized 18 h after the LPS injection. The other animals in the experiment were monitored for survival for 3 days (72 h). Serum samples were collected at baseline and at euthanasia and used to measure the levels of cytokines and other inflammatory markers (CRP) using a Bioplex mouse cytokine assay or ELISAs. Created with BioRender.

Mice were allotted into six groups comprising 15 animals each (Fig. 8). Animals were housed in a pathogen-free environment maintained at 22°C with 50%–60% humidity and a 12-h light/dark cycle. The mice were provided with a standard pellet diet and water *ad libitum*. On the day of the experiment, animals from groups 1 to 4 received an intraperitoneal injection of a lethal dose of 12 mg/kg BW LPS (*E. coli* 0111: B4); LPS dissolved in sterile distilled water was administered via i.p. injection (10, 43). One hour after the LPS injection, animals from groups 1, 2, and 3 were treated with a single i.p. injection containing 50, 100, or 200 μg Fh15 dose, respectively, prepared in sterile endotoxin-free PBS. Group 4 was treated with placebo (equivalent PBS volume). Control groups received a single i.p. injection with a 100 μg Fh15 dose or a single i.p. injection with equivalent PBS volume. Animals were returned to their respective cage and closely monitored for mortality every hour during the first 12 h following the LPS injection and then every 4 h until the end of the experiment. At humane 18 h following the LPS challenge, five animals from each experimental group were randomly selected for bled out, which was performed by terminal cardiac puncture under deep anesthesia. Next, animals were euthanized by cervical dislocation. The other animals remained in their respective cages and were monitored every 4 h until a humane endpoint of 72 h. At this time, all animals were bled out and euthanized as described above. During monitoring, any animal exhibiting signs such as inability to maintain an upright posture, difficulty in feeding or drinking, anorexia, clinical dehydration, labored or agonal breathing, cyanosis, or unconsciousness with no response to external stimuli was immediately anesthetized for blood collection and euthanasia, as described above. The survival of animals was carefully monitored for 72 h and evaluated using Kaplan–Meier survival curves.

### Mouse primary cells isolation and differentiation

BMDMs were isolated from the femoral and tibial shafts of naïve mice using a previously established protocol (6, 8, 44). Briefly, femoral shafts of mice were flushed with 3 mL of cold sterile PBS to obtain a cell suspension, which was then sieved to eliminate large clumps. Cells were washed three times with sterile complete RPMI-1640 medium supplemented with 20 mM L-glutamine, 10 mM HEPES, 10% (vol/vol) of iFBS, 100 U/mL penicillin, and 100 μg/mL of streptomycin. Cells were adjusted to 1 × 10^5^ cells per well with differentiation medium (complete RPMI-1640 supplemented with 20 ng/mL M-CSF from R&D Systems Ltd., Minneapolis, MN, USA) and cultured in 24-well NuncTM Multidishes Up-Cell Surface plate (Thermo Fisher, Waltham, MA, USA) at 37°C, 5% CO_2_. On day 3, non-adherent cells were removed, and the adherent cells were replenished with fresh differentiation medium. Cells' incubation was prolonged for 7 days to allow full macrophage maturation. For experiments, BMDMs were seeded into 24-well plates (NuncTM Multidishes Up-Cell Surface) at 1 × 10^6^ cells per well in complete RPMI-1640 medium and then treated in triplicate with Fh15 (10 μg/mL) for 30 min prior to stimulation with 100 ng/mL LPS (Fh15 + LPS). Cells treated only with Fh15 (10 μg/mL), LPS (100 ng/mL), or PBS were used as controls.

### Cytokine measurement by BioPlex

Serum samples collected from mice were diluted fourfold in sample buffer and tested in duplicate in a multiplex bead-based immunoassay (Bio-plex Pro Mouse cytokine Th1/Th2 8-plex Bio-Rad, Hercules, CA) using the Luminex MAGPIX system. Data were analyzed with Bio-Plex Manager 6.1 software (Bio-Rad, Hercules, CA) using a 5-parameter logistic standard curve.

### Measurement of serum interleukin-37 and C-reactive protein levels in mice

Levels of IL-37 present in the serum of animals subjected to the challenge described above were quantified using a mouse IL-37 ELISA kit (My BioSource, San Diego, CA) following the manufacturer’s instructions. Serum samples were diluted twofold prior to testing, and IL-37 concentrations were determined by comparison with the corresponding standard curves. Similarly, serum levels of CRP were quantified using a mouse CRP ELISA kit (Thermo Fisher, Waltham, MA, USA) according to the manufacturer’s instructions. Serum samples were diluted 100-fold, and the CRP concentrations were calculated by comparison with the corresponding 4-parameter logistic (4PL) standard curve.

### RNA isolation and RT-qPCR analysis

Total RNA was extracted from BMDMs using an AllPrep DNA/RNA/Protein Mini Kit (Qiagen, Hilden, Germany). RNA concentration was measured with a Nanodrop 1000 spectrophotometer (Thermo Fisher Scientific, Waltham, MA, USA). cDNA synthesis and amplification of genes encoding TNF-α, NOS2, IL-10, CD36, Fizz1, and Ym-1 were performed in triplicate using the Power SYBR Green RNA-to-CT 1-Step Kit (Applied Biosystems, Waltham, MA, USA) on the QuantStudio 3 PCR system (Applied Biosystems). For each reaction, 100 ng of RNA was used. The thermal cycling protocol included an initial step at 48°C for 30 min, followed by 95°C for 10 min, and then 40 cycles comprising 95°C for 15 s and 60°C for 1 min. The primers used for each gene are listed in Table 2. Primer’s concentration was optimized, and dissociation curves were generated for each primer set to confirm the specificity of amplification. The 2^−ΔΔCt^ method (45) was used to quantify relative gene expression using GAPDH as a housekeeping gene, and the results were expressed as fold change relative to expression in the cells stimulated with PBS.

**TABLE 2:** RT-qPCR primers

Gene	Primers	Sequence 5′−3′
GAPDH	Forward	CATGGCCTTCCGTGTTCCTA
	Reverse	CCTGCTTCACCACCTTCTTGAT
NOS2	Forward	TTCACCCAGTTGTGCATCGACCTA
	Reverse	TCCATGGTCACCTCCAACACAAGA
TNF-α	Forward	AAGCCTGTAGCCCACGTCGTA
	Reverse	AGGTACAACCCATCGGCTGG
IL-10	Forward	ATTTGAATTCCCTGGGTGAGAAG
	Reverse	CACAGGGGAGAAATCGATGACA
Fizz1	Forward	TACTTGCAACTGCCTGTGCTTACT
	Reverse	TATCAAAGCTGGGTTCTCCACCTC
CD206	Forward	AAACACAGACTGACCCTTCCC
	Reverse	GTTAGTGTACCGCACCCTCC
CD14	Forward	TTGAACCTCCGCAACGTGTCGT
	Reverse	CGCAGGAAAAGTTGAGCGAGTG

### Flow cytometry analysis

BMDMs treated overnight at 37°C, 5% CO_2_ with PBS, LPS, IL-4, Fh15, or a combination of Fh15 and LPS as described above, were transferred at room temperature to harvest cells. Cells were suspended in sterile PBS and after being adjusted to 5 × 10^5^ cells were stained with an antibody cocktail containing Fc-block (1:500) and live/dead Aqua Zoombie (1:400). After an incubation of 30 min at room temperature in the dark, cells were stained with specific antibodies against CD11b FITC (1:200), F4/80 PE-CF594 (1:200), iNOS PE (1:200), CD80 BV711 (1:400), and CD38 PerCP/Cy5.5 (1:200) (Table 3). After an incubation of 30 min at 4°C in the dark, cells were washed with FACS buffer, fixed for 10 min at 4°C with fixation buffer (BD Biosciences, Franklin Lakes, NJ, USA), and subsequently centrifuged at 1,200 rpm for 5 min. Unlabeled or isotype-matched stained cells were used as controls. Dead cells were excluded from analysis, and macrophages were identified based on the F4/80+ CD11b+ marker profile. Data acquisition was carried out using a Miltenyi MACSQuant Analyzer 10, and the resulting data were analyzed with FlowJo software (FlowJo, LLC). The gating strategy is shown in Fig. S1.

**TABLE 3:** Antibodies cocktail used in the flow cytometry analysis

Cell marker	Labeling	Clone	Source	Catalog number
F4/80	PE-CF594	T45-2342	BD	565613
CD11b	FITC	M1/70	Biolegend	101206
CD80	Brilliant Violet 711	16-10A1	Biolegend	104743
NOS2	PE	CXNFT	eBioscience	12-5920-82
CD38	PerCP/Cy5.5	90	Biolegend	102721

### Phagocytosis assay

The phagocytic ability of BMDM in the presence of Fh15 was measured using a Phagocytosis Assay Kit that uses a green *E. coli* labeled with fluorescein. Briefly, BMDM were plated at 1.0 × 10^6^ viable cells/well in a 96-well plate and treated for 30 min with 100 ng of Fh15. After the incubation, cells were then treated with 5 µL of a fluorescent *E. coli* suspension and incubated at 5% CO_2_, 37°C for 6 h. After incubation, the *E. coli* suspension was removed by aspiration, which was followed by the addition of 300 µL/well of ice-cold assay buffer XXVII/Phagocytosis assay buffer. The microplate was read at 490 nm excitation and 520 nm emission. The net phagocytosis percentage and the response of the phagocytosis effector agent were calculated by calculating the net experimental reading and the net positive reading according to the manufacturer’s instructions.

### Statistical analysis

Each *in vivo* experiment was replicated twice with 15 animals per group. Each serum cytokine, or CRP determination, as well as every *in vitro* assay, was performed in duplicate, and data that satisfied a normal distribution were presented as mean values and standard error of the mean (±SEM) from at least three independent experiments. Statistical significance (*P* < 0.05, two-tailed) for each variable was estimated using the statistical package of GraphPad Prism version 10 (GraphPad Software, Boston, MA, USA).

Survival data were analyzed using Kaplan–Meier survival curves, and differences between groups were assessed with the log-rank (Mantel–Cox) test. For multiple pairwise comparisons, *P* values were adjusted using the Holm–Šídák method. All other data were analyzed using one-way ANOVA, followed by Tukey’s multiple-comparisons test. Data are presented as mean ± SEM or SD, as indicated in the figure legends. Statistical significance was defined as *P* < 0.05. Analyses were performed using GraphPad Prism version 10.

### Study limitations

The study has potential limitations that should be acknowledged. Although macroscopic evaluation of the abdominal cavity in animals that survived LPS challenge, along with a scoring system during monitoring and serological assessment of inflammatory markers, allowed us to determine the effectiveness of Fh15 treatment, the integration of histopathological analysis would have strengthened these findings. In particular, microscopic examination of major organs commonly affected during septic shock, such as kidneys, lungs, and liver, would have provided critical insight into tissue-level damage and protection. Additionally, a longer post-challenge follow-up period (e.g., for 7 or 10 days) would also be necessary to confirm sustained survival and to rule out delayed mortality due to relapse of disease. Moreover, it will be necessary to study the effect of Fh15 on other immune cell populations, particularly neutrophils, which play essential roles during early and critical phases of endotoxin-induced sepsis and can significantly influence survival. Although these aspects could not be addressed in the present study due to economic limitations, they are integral components of ongoing and future studies aimed at fully defining the therapeutic potential of Fh15.

## SUPPLEMENTAL MATERIAL

Supplemental material spectrum.02756-25-s0001.pdf Fig. S1; Table S1.

ASM does not own the copyrights to Supplemental Material that may be linked to, or accessed through, an article. The authors have granted ASM a non-exclusive, world-wide license to publish the Supplemental Material files. Please contact the corresponding author directly for reuse.
