# Supplementary material for: Protection of mice against septic shock induced by lethal intraperitoneal dose of LPS by a recombinant Fasciola hepatica fatty acid binding protein
Source: Microbiol Spectr. 2026 Feb 27;14(4):e02756-25. doi: 10.1128/spectrum.02756-25 (PMC13055263; doi:10.1128/spectrum.02756-25)
Supplement: Supplemental material — Fig. S1; Table S1. [file spectrum.02756-25-s0001.pdf]

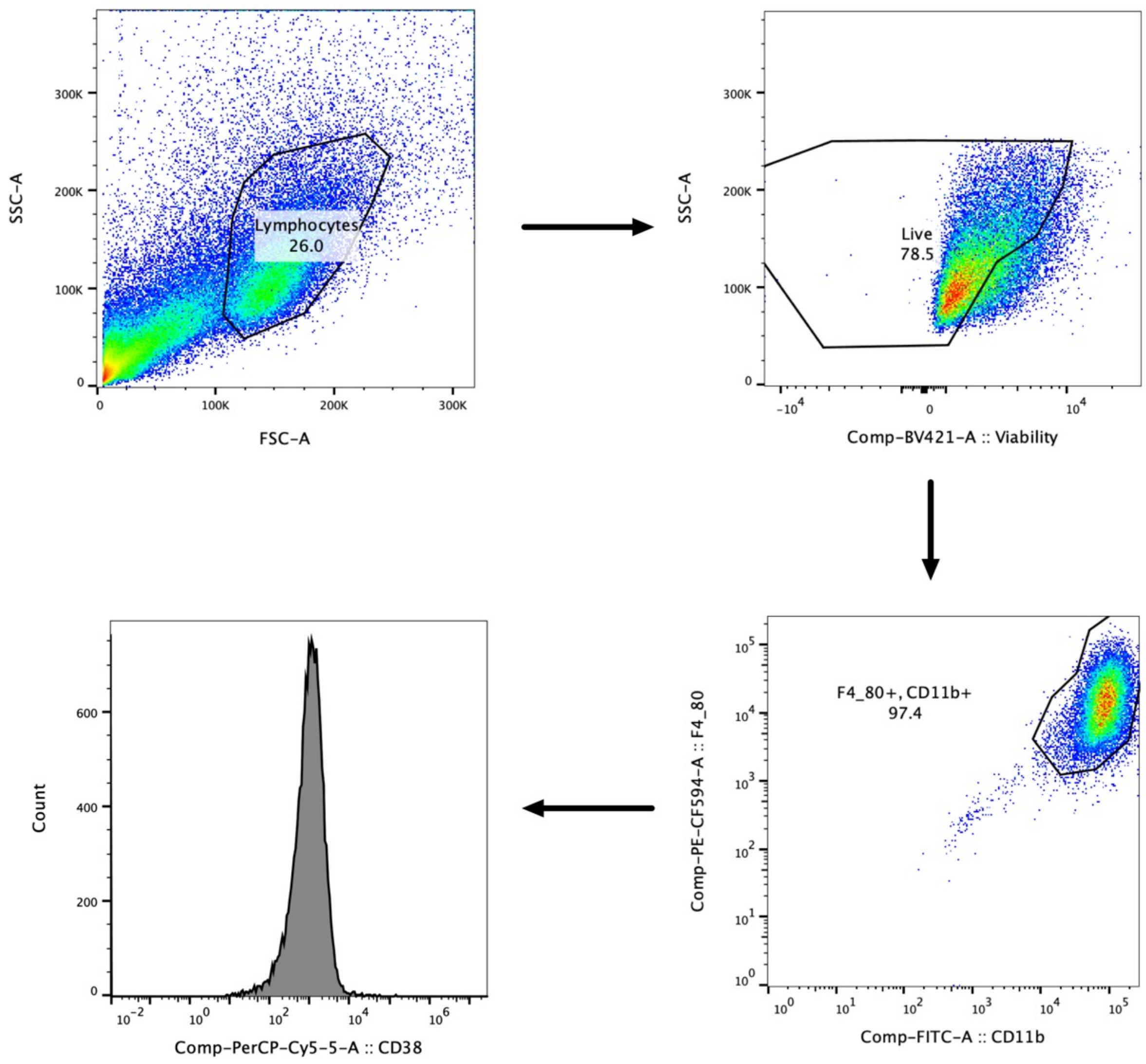

Figure-S1. Schematic representation of the gating strategy for flow cytometry. Bone marrow derived macrophages (BMDM) were isolated and differentiate. A single-cell suspensions of 5×10^5 cells per well were stained using a 10-color antibody panel for multiparametric flow cytometric analysis. Cells were incubated with an antibody cocktail containing Fc-block, live/dead Aqua Zoombie and then stained with antibodies against F4/80, CD11b, CD38, CD80, and NOS2.

Table S1. Criteria used to determine the Murine Sepsis Score (MSS). Monitoring of the animals was conducted by two investigators during every hour after induction of septic shock by intraperitoneal (i.p.) injection of a lethal dose of LPS (12mg/kg body weight) for 12 hours and then every four hours until the end of the experiment (72 hours after the LPS injection). Mice were evaluated while they were still in their cages (with the lids removed for better visualization). The MSS system evaluates disease severity based on appearance, level of consciousness, activity, response to stimuli, eyes and respiration rate and respiration quality. Each of these variables are given a score between 0 and 4 with higher scores indicating more severe clinical impairment. Mice with MSS at any given time point greater than 25 or with points ascribed to respiratory rate or quality increased by more than 3 died or were sacrificed. At the conclusion of the experiments animals that survived to the challenge were sacrificed. Each experimental group consisted of 15 animals allotted into cage of 5 animals each and the study was conducted independently over a period of seven months. The 'x' in the table indicates: parameter no determined because the animal died or was sacrificed. This monitoring chart was adapted from the study published by Shrum, B et al. *BMC Research Notes* 2014, 7:233. http://www.biomedcentral.com/1756-0500/7/233.

Monitor Chart
Group: LPS (12 mg/ kg BW)
Date of injection with LPS- *E. coli*: May 13, 2021
Time of injection: 4:00 pm
Cage: #1 (n=5)

Variable	1h					2h					3h					4h					5h					6h					7h					8h					9h				
Appearance	0	0	0	0	0	0	0	0	0	0	0	0	0	0	0	0	0	0	0	0	0	0	0	0	0	0	0	0	0	0	0	0	0	0	0	1	1	1	1	1	2	1	2	2	2
Level of consciousness	0	0	0	0	0	0	0	0	0	0	0	0	0	0	0	0	0	0	0	0	0	0	0	0	0	0	0	0	0	0	0	0	0	0	0	0	0	0	0	0	1	1	1	1	1
Activity	0	0	0	0	0	0	0	0	0	0	0	0	0	0	0	0	0	0	0	0	0	0	0	0	0	0	0	0	0	0	0	0	0	0	0	0	0	0	0	0	1	2	1	1	1
Responses to stimulus	0	0	0	0	0	0	0	0	0	0	0	0	0	0	0	0	0	0	0	0	0	0	0	0	0	0	0	0	0	0	0	0	0	0	0	0	0	0	0	0	1	1	1	1	1
Eyes	0	0	0	0	0	0	0	0	0	0	0	0	0	0	0	0	0	0	0	0	0	0	0	0	0	0	0	0	0	0	0	0	0	0	0	1	1	1	1	1	1	1	2	1	1

Respiration rate	0	0	0	0	0	0	0	0	0	0	0	0	0	0	0	0	0	0	0	0	0	0	0	0	0	0	0	0	0	0	0	0	0	0	0	1	1	0	1	1	1	1	1	2	2
Respiration quality	0	0	0	0	0	0	0	0	0	0	0	0	0	0	0	0	0	0	0	0	0	0	0	0	0	0	0	0	0	0	0	0	0	0	0	0	0	0	0	0	0	0	0	0	0
**Total**	0	0	0	0	0	0	0	0	0	0	0	0	0	0	0	0	0	0	0	0	0	0	0	0	0	0	0	0	0	0	0	0	0	0	0	3	3	2	3	3	7	7	8	8	8

Table S1 (Continuation)
Monitor Chart
Group: LPS (12 mg/ kg BW)
Date of injection with LPS- *E. coli*: May 13, 2021
Time of injection: 4:00 pm
Cage: #1 (n=5)

Variable	10h					11h					12h					16h					20h					24h					28h					32h				
Appearance	2	2	2	2	2	3	2	2	2	2	3	3	3	3	3	3	4	4	4	4	4	4	4	X	X	X	X	X	X	X	X	X	X	X	X	X	X	X	X	X
Level of consciousness	2	2	1	1	2	2	2	1	2	1	3	3	3	3	3	3	3	3	4	4	4	4	4	X	X	X	X	X	X	X	X	X	X	X	X	X	X	X	X	X
Activity	2	2	2	2	1	2	2	2	2	2	3	3	2	3	3	3	3	3	4	4	4	4	4	X	X	X	X	X	X	X	X	X	X	X	X	X	X	X	X	X
Responses to stimulus	2	1	1	1	1	2	2	1	1	1	2	2	2	2	2	3	3	3	3	4	4	4	4	X	X	X	X	X	X	X	X	X	X	X	X	X	X	X	X	X
Eyes	2	2	2	2	2	2	2	2	2	2	2	2	2	2	2	3	3	3	3	4	4	4	4	X	X	X	X	X	X	X	X	X	X	X	X	X	X	X	X	X
Respiration rate	2	2	2	2	2	2	2	2	2	2	3	3	2	2	2	3	3	3	3	4	4	4	4	X	X	X	X	X	X	X	X	X	X	X	X	X	X	X	X	X
Respiration quality	1	1	0	1	0	1	1	1	1	1	2	1	1	1	1	3	3	3	3	4	4	4	4	X	X	X	X	X	X	X	X	X	X	X	X	X	X	X	X	X
**Total**	13	12	10	11	10	14	13	11	12	11	18	17	15	16	16	21	22	22	24	28	28	28	28	X	X	X	X	X	X	X	X	X	X	X	X	X	X	X	X	X

Table S1 (Continuation)
Monitor Chart
Group: LPS (12 mg/ kg BW)
Date of injection with LPS- *E. coli*: May 13, 2021
Time of injection: 4:00 pm
Cage: #1 (n=5)

Variable	36h					40h					44h					48					52h					56h					60h					72h				
Appearance	x	x	x	x	x	x	x	x	x	x	x	x	x	x	x	x	x	x	x	x	x	x	x	x	x	x	x	x	x	x	x	x	x	x	x	x	x	x	x	x
Level of consciousness	x	x	x	x	x	x	x	x	x	x	x	x	x	x	x	x	x	x	x	x	x	x	x	x	x	x	x	x	x	x	x	x	x	x	x	x	x	x	x	x
Activity	x	x	x	x	x	x	x	x	x	x	x	x	x	x	x	x	x	x	x	x	x	x	x	x	x	x	x	x	x	x	x	x	x	x	x	x	x	x	x	x
Responses to stimulus	x	x	x	x	x	x	x	x	x	x	x	x	x	x	x	x	x	x	x	x	x	x	x	x	x	x	x	x	x	x	x	x	x	x	x	x	x	x	x	x
Eyes	x	x	x	x	x	x	x	x	x	x	x	x	x	x	x	x	x	x	x	x	x	x	x	x	x	x	x	x	x	x	x	x	x	x	x	x	x	x	x	x
Respiration rate	x	x	x	x	x	x	x	x	x	x	x	x	x	x	x	x	x	x	x	x	x	x	x	x	x	x	x	x	x	x	x	x	x	x	x	x	x	x	x	x
Respiration quality	x	x	x	x	x	x	x	x	x	x	x	x	x	x	x	x	x	x	x	x	x	x	x	x	x	x	x	x	x	x	x	x	x	x	x	x	x	x	x	x
**Total**	x	x	x	x	x	x	x	x	x	x	x	x	x	x	x	x	x	x	x	x	x	x	x	x	x	x	x	x	x	x	x	x	x	x	x	x	x	x	x	x

Table S1 (Continuation)
Monitor Chart
Group: LPS (12 mg/ kg BW)
Date of injection with LPS- *E. coli*: May 13, 2021
Time of injection: 4:15 pm
Cage: #2 (n=5)

Variable	1h					2h					3h					4h					5h					6h					7h					8h					9h				
Appearance	0	0	0	0	0	0	0	0	0	0	0	0	0	0	0	0	0	0	0	0	0	0	0	0	0	0	0	0	0	0	0	0	0	0	0	1	1	1	1	1	2	2	1	1	2
Level of consciousness	0	0	0	0	0	0	0	0	0	0	0	0	0	0	0	0	0	0	0	0	0	0	0	0	0	0	0	0	0	0	0	0	0	0	0	0	0	0	0	0	1	1	1	1	1
Activity	0	0	0	0	0	0	0	0	0	0	0	0	0	0	0	0	0	0	0	0	0	0	0	0	0	0	0	0	0	0	0	0	0	0	0	0	0	0	0	0	1	1	2	2	1
Responses to stimulus	0	0	0	0	0	0	0	0	0	0	0	0	0	0	0	0	0	0	0	0	0	0	0	0	0	0	0	0	0	0	0	0	0	0	0	0	0	0	0	0	1	1	1	1	1
Eyes	0	0	0	0	0	0	0	0	0	0	0	0	0	0	0	0	0	0	0	0	0	0	0	0	0	0	0	0	0	0	0	0	0	0	0	1	0	1	1	0	1	1	1	1	1
Respiration rate	0	0	0	0	0	0	0	0	0	0	0	0	0	0	0	0	0	0	0	0	0	0	0	0	0	0	0	0	0	0	0	0	0	0	0	0	0	0	0	0	1	1	1	1	1
Respiration quality	0	0	0	0	0	0	0	0	0	0	0	0	0	0	0	0	0	0	0	0	0	0	0	0	0	0	0	0	0	0	0	0	0	0	0	0	0	0	0	0	0	0	0	0	0
**Total**	0	0	0	0	0	0	0	0	0	0	0	0	0	0	0	0	0	0	0	0	0	0	0	0	0	0	0	0	0	0	0	0	0	0	0	2	1	2	2	1	7	7	7	7	7

Table S1 (Continuation)
Monitor Chart
Group: LPS (12 mg/ kg BW)
Date of injection with LPS- *E. coli*: May 13, 2021
Time of injection: 4:15 pm
Cage: #2 (n=5)

Variable	10h					11h					12h					16h					20h					24h					28h					32h				
Appearance	2	2	2	2	2	3	2	2	3	2	3	3	3	3	4	4	4	4	4	X	4	4	4	X	X	X	X	X	X	X	X	X	X	X	X	X	X	X	X	X
Level of consciousness	2	2	1	2	2	2	2	2	2	2	3	3	3	3	4	4	4	4	4	X	4	4	4	X	X	X	X	X	X	X	X	X	X	X	X	X	X	X	X	X
Activity	2	2	2	2	1	2	3	2	2	2	3	3	2	3	4	4	4	4	4	X	4	4	4	X	X	X	X	X	X	X	X	X	X	X	X	X	X	X	X	X
Responses to stimulus	1	2	1	1	1	2	2	2	2	2	3	2	3	3	4	4	4	4	3	X	4	4	4	X	X	X	X	X	X	X	X	X	X	X	X	X	X	X	X	X
Eyes	2	2	2	2	2	2	2	2	2	2	2	3	3	3	4	4	4	4	4	X	4	4	4	X	X	X	X	X	X	X	X	X	X	X	X	X	X	X	X	X
Respiration rate	2	2	2	2	2	2	2	2	2	2	3	3	2	3	4	4	4	4	3	X	4	4	4	X	X	X	X	X	X	X	X	X	X	X	X	X	X	X	X	X
Respiration quality	1	1	1	1	0	1	1	1	1	1	2	2	2	2	3	4	4	4	3	X	4	4	4	X	X	X	X	X	X	X	X	X	X	X	X	X	X	X	X	X
**Total**	12	13	11	12	10	14	14	13	14	13	19	19	18	20	27	28	28	28	28	X	28	28	28	X	X	X	X	X	X	X	X	X	X	X	X	X	X	X	X	X

Table S1 (Continuation)

Monitor Chart

Group: LPS (12 mg/ kg BW)

Date of injection with LPS- *E. coli*: May 13, 2021

Time of injection: 4:15 pm

Cage: #2 (n=5)

Variable	36h					40h					44h					48					52h					56h					60h					72h				
Appearance	x	x	x	x	x	x	x	x	x	x	x	x	x	x	x	x	x	x	x	x	x	x	x	x	x	x	x	x	x	x	x	x	x	x	x	x	x	x	x	x
Level of consciousness	x	x	x	x	x	x	x	x	x	x	x	x	x	x	x	x	x	x	x	x	x	x	x	x	x	x	x	x	x	x	x	x	x	x	x	x	x	x	x	x
Activity	x	x	x	x	x	x	x	x	x	x	x	x	x	x	x	x	x	x	x	x	x	x	x	x	x	x	x	x	x	x	x	x	x	x	x	x	x	x	x	x
Responses to stimulus	x	x	x	x	x	x	x	x	x	x	x	x	x	x	x	x	x	x	x	x	x	x	x	x	x	x	x	x	x	x	x	x	x	x	x	x	x	x	x	x
Eyes	x	x	x	x	x	x	x	x	x	x	x	x	x	x	x	x	x	x	x	x	x	x	x	x	x	x	x	x	x	x	x	x	x	x	x	x	x	x	x	x
Respiration rate	x	x	x	x	x	x	x	x	x	x	x	x	x	x	x	x	x	x	x	x	x	x	x	x	x	x	x	x	x	x	x	x	x	x	x	x	x	x	x	x
Respiration quality	x	x	x	x	x	x	x	x	x	x	x	x	x	x	x	x	x	x	x	x	x	x	x	x	x	x	x	x	x	x	x	x	x	x	x	x	x	x	x	x
**Total**	x	x	x	x	x	x	x	x	x	x	x	x	x	x	x	x	x	x	x	x	x	x	x	x	x	x	x	x	x	x	x	x	x	x	x	x	x	x	x	x

Table S1 (Continuation)

Monitor Chart

Group: LPS (12 mg/ kg BW)

Date of injection with LPS- *E. coli*: December 15, 2022

Time of injection: 4:50 pm

Cage: #1 (n=5)

Variable	1h					2h					3h					4h					5h					6h					7h					8h					9h				
Appearance	0	0	0	0	0	0	0	0	0	0	0	0	0	0	0	0	0	0	0	0	0	0	0	0	0	0	0	0	0	0	0	0	0	0	0	1	1	1	1	1	2	2	2	2	2
Level of consciousness	0	0	0	0	0	0	0	0	0	0	0	0	0	0	0	0	0	0	0	0	0	0	0	0	0	0	0	0	0	0	0	0	0	0	0	0	0	0	0	0	1	1	1	1	1
Activity	0	0	0	0	0	0	0	0	0	0	0	0	0	0	0	0	0	0	0	0	0	0	0	0	0	0	0	0	0	0	0	0	0	0	0	0	0	0	0	0	1	1	2	1	1
Responses to stimulus	0	0	0	0	0	0	0	0	0	0	0	0	0	0	0	0	0	0	0	0	0	0	0	0	0	0	0	0	0	0	0	0	0	0	0	0	0	0	0	0	1	1	1	1	1
Eyes	0	0	0	0	0	0	0	0	0	0	0	0	0	0	0	0	0	0	0	0	0	0	0	0	0	0	0	0	0	0	0	0	0	0	0	1	1	1	1	1	1	2	2	2	2
Respiration rate	0	0	0	0	0	0	0	0	0	0	0	0	0	0	0	0	0	0	0	0	0	0	0	0	0	0	0	0	0	0	0	0	0	0	0	1	0	0	1	1	1	2	1	2	2
Respiration quality	0	0	0	0	0	0	0	0	0	0	0	0	0	0	0	0	0	0	0	0	0	0	0	0	0	0	0	0	0	0	0	0	0	0	0	0	0	0	0	0	0	0	0	0	0
**Total**	0	0	0	0	0	0	0	0	0	0	0	0	0	0	0	0	0	0	0	0	0	0	0	0	0	0	0	0	0	0	0	0	0	0	0	3	2	2	3	3	7	9	9	9	9

Table S1 (Continuation)

Monitor Chart

Group: LPS (12 mg/ kg BW)

Date of injection with LPS- *E. coli*: December 15, 2022

Time of injection: 4:50 pm

Cage: #1 (n=5)

Variable	10h					11h					12h					16h					20h					24h					28h					32h				
Appearance	3	2	2	2	3	3	3	2	2	3	3	3	3	4	4	4	4	4	X	X	4	4	4	X	X	X	X	X	X	X	X	X	X	X	X	X	X	X	X	X
Level of consciousness	2	2	1	1	2	2	2	2	2	2	3	3	3	4	4	3	3	3	X	X	4	4	4	X	X	X	X	X	X	X	X	X	X	X	X	X	X	X	X	X
Activity	2	2	2	2	3	3	3	2	2	2	3	3	3	4	4	4	4	4	X	X	4	4	4	X	X	X	X	X	X	X	X	X	X	X	X	X	X	X	X	X
Responses to stimulus	2	2	2	1	1	2	2	2	2	2	3	3	3	4	4	4	4	4	X	X	4	4	4	X	X	X	X	X	X	X	X	X	X	X	X	X	X	X	X	X
Eyes	2	2	2	2	2	3	2	2	2	2	3	3	3	4	4	4	4	4	X	X	4	4	4	X	X	X	X	X	X	X	X	X	X	X	X	X	X	X	X	X
Respiration rate	2	2	2	2	2	2	2	2	2	2	3	3	3	4	4	3	4	3	X	X	4	4	4	X	X	X	X	X	X	X	X	X	X	X	X	X	X	X	X	X
Respiration quality	1	1	1	1	1	2	1	2	2	2	3	3	2	3	4	3	3	3	X	X	3	4	4	X	X	X	X	X	X	X	X	X	X	X	X	X	X	X	X	X
**Total**	14	13	12	11	14	17	15	14	14	15	21	21	20	27	28	25	26	25	X	X	27	28	28	X	X	X	X	X	X	X	X	X	X	X	X	X	X	X	X	X

Table S1 (Continuation)
Monitor Chart
Group: LPS (12 mg/ kg BW)
Date of injection with LPS- *E. coli*: December 15, 2022
Time of injection: 4:50 pm
Cage: #1 (n=5)

Variable	36h					40h					44h					48					52h					56h					60h					72h				
Appearance	x	x	x	x	x	x	x	x	x	x	x	x	x	x	x	x	x	x	x	x	x	x	x	x	x	x	x	x	x	x	x	x	x	x	x	x	x	x	x	x
Level of consciousness	x	x	x	x	x	x	x	x	x	x	x	x	x	x	x	x	x	x	x	x	x	x	x	x	x	x	x	x	x	x	x	x	x	x	x	x	x	x	x	x
Activity	x	x	x	x	x	x	x	x	x	x	x	x	x	x	x	x	x	x	x	x	x	x	x	x	x	x	x	x	x	x	x	x	x	x	x	x	x	x	x	x
Responses to stimulus	x	x	x	x	x	x	x	x	x	x	x	x	x	x	x	x	x	x	x	x	x	x	x	x	x	x	x	x	x	x	x	x	x	x	x	x	x	x	x	x
Eyes	x	x	x	x	x	x	x	x	x	x	x	x	x	x	x	x	x	x	x	x	x	x	x	x	x	x	x	x	x	x	x	x	x	x	x	x	x	x	x	x
Respiration rate	x	x	x	x	x	x	x	x	x	x	x	x	x	x	x	x	x	x	x	x	x	x	x	x	x	x	x	x	x	x	x	x	x	x	x	x	x	x	x	x
Respiration quality	x	x	x	x	x	x	x	x	x	x	x	x	x	x	x	x	x	x	x	x	x	x	x	x	x	x	x	x	x	x	x	x	x	x	x	x	x	x	x	x
**Total**	x	x	x	x	x	x	x	x	x	x	x	x	x	x	x	x	x	x	x	x	x	x	x	x	x	x	x	x	x	x	x	x	x	x	x	x	x	x	x	x

Table S1 (Continuation)
Monitor Chart
Group: LPS (12 mg/ kg BW) + 50µg Fh15 (~ 2.5 mg/kg BW)
Date of injection with LPS- *E. coli*: May 13, 2021
Time of injection: 5:00 pm
Cage: #1 (n=5)

Variable	1h					2h					3h					4h					5h					6h					7h					8h					9h				
Appearance	0	0	0	0	0	0	0	0	0	0	0	0	0	0	0	0	0	0	0	0	0	0	0	0	0	0	0	0	0	0	0	0	0	0	0	1	1	1	1	1	1	1	1	1	1
Level of consciousness	0	0	0	0	0	0	0	0	0	0	0	0	0	0	0	0	0	0	0	0	0	0	0	0	0	0	0	0	0	0	0	0	0	0	0	0	0	0	0	0	0	1	1	1	0
Activity	0	0	0	0	0	0	0	0	0	0	0	0	0	0	0	0	0	0	0	0	0	0	0	0	0	0	0	0	0	0	0	0	0	0	0	0	0	1	0	0	1	1	1	1	1
Responses to stimulus	0	0	0	0	0	0	0	0	0	0	0	0	0	0	0	0	0	0	0	0	0	0	0	0	0	0	0	0	0	0	0	0	0	0	0	0	0	0	0	0	1	1	1	1	1
Eyes	0	0	0	0	0	0	0	0	0	0	0	0	0	0	0	0	0	0	0	0	0	0	0	0	0	0	0	0	0	0	0	0	0	0	0	1	1	1	1	1	1	1	1	1	1
Respiration rate	0	0	0	0	0	0	0	0	0	0	0	0	0	0	0	0	0	0	0	0	0	0	0	0	0	0	0	0	0	0	0	0	0	0	0	0	1	0	0	0	1	1	1	0	1
Respiration quality	0	0	0	0	0	0	0	0	0	0	0	0	0	0	0	0	0	0	0	0	0	0	0	0	0	0	0	0	0	0	0	0	0	0	0	0	0	0	0	0	0	0	0	0	0
**Total**	0	0	0	0	0	0	0	0	0	0	0	0	0	0	0	0	0	0	0	0	0	0	0	0	0	0	0	0	0	0	0	0	0	0	0	2	3	3	1	2	5	6	6	4	3

Table S1 (Continuation)

Monitor Chart

Group: LPS (12 mg/ kg BW) + 50µg Fh15 (~ 2.5 mg/kg BW)

Date of injection with LPS- *E. coli*: May 13, 2021

Time of injection: 5:00 pm

Cage: #1 (n=5)

Variable	10h					11h					12h					16h					20h					24h					28h					32h				
Appearance	2	2	2	2	2	2	2	2	2	2	3	2	3	2	3	3	3	3	3	3	4	3	4	4	4	4	4	4	2	2	X	X	X	1	1	X	X	X	0	0
Level of consciousness	1	1	1	1	1	1	2	1	2	2	2	2	2	2	2	2	2	3	3	3	3	3	3	3	3	3	3	3	2	1	X	X	X	0	0	X	X	X	0	0
Activity	1	1	1	1	1	2	2	2	2	2	2	2	3	2	2	3	3	3	3	3	3	3	4	3	4	4	4	4	2	2	X	X	X	1	1	X	X	X	0	0
Responses to stimulus	1	2	2	2	2	2	2	2	2	2	2	2	2	2	2	3	2	3	3	3	3	3	3	3	3	4	3	4	1	1	X	X	X	0	0	X	X	X	0	0
Eyes	2	2	2	2	2	2	2	2	2	2	2	2	2	2	2	3	2	3	3	3	3	3	3	3	4	4	4	4	2	2	X	X	X	1	1	X	X	X	0	0
Respiration rate	1	1	1	1	1	1	1	2	2	2	2	2	2	2	2	2	2	2	3	3	3	3	3	3	4	4	4	4	2	1	X	X	X	1	0	X	X	X	0	0
Respiration quality	0	1	1	0	1	1	1	1	1	1	2	2	2	2	2	2	2	2	2	3	3	2	3	3	3	3	3	3	1	1	X	X	X	0	0	X	X	X	0	0
**Total**	8	10	10	8	8	11	12	12	12	12	15	14	16	14	14	18	16	19	18	16	22	20	23	22	20	26	25	26	12	10	X	X	X	4	3	X	X	X	0	0

Table S1 (Continuation)
Monitor Chart
Group: LPS (12 mg/ kg BW) + 50µg Fh15 (~ 2.5 mg/kg BW)
Date of injection with LPS- *E. coli*: May 13, 2021
Time of injection: 5:00 pm
Cage: #1 (n=5)

Variable	36h					40h					44h					48					52h					56h					60h					72h				
Appearance	X	X	X	0	0	X	X	X	0	0	X	X	X	0	0	X	X	X	0	0	X	X	X	0	0	X	X	X	0	0	X	X	X	0	0	X	X	X	0	0
Level of consciousness	X	X	X	0	0	X	X	X	0	0	X	X	X	0	0	X	X	X	0	0	X	X	X	0	0	X	X	X	0	0	X	X	X	0	0	X	X	X	0	0
Activity	X	X	X	0	0	X	X	X	0	0	X	X	X	0	0	X	X	X	0	0	X	X	X	0	0	X	X	X	0	0	X	X	X	0	0	X	X	X	0	0
Responses to stimulus	X	X	X	0	0	X	X	X	0	0	X	X	X	0	0	X	X	X	0	0	X	X	X	0	0	X	X	X	0	0	X	X	X	0	0	X	X	X	0	0
Eyes	X	X	X	0	0	X	X	X	0	0	X	X	X	0	0	X	X	X	0	0	X	X	X	0	0	X	X	X	0	0	X	X	X	0	0	X	X	X	0	0
Respiration rate	X	X	X	0	0	X	X	X	0	0	X	X	X	0	0	X	X	X	0	0	X	X	X	0	0	X	X	X	0	0	X	X	X	0	0	X	X	X	0	0
Respiration quality	X	X	X	0	0	X	X	X	0	0	X	X	X	0	0	X	X	X	0	0	X	X	X	0	0	X	X	X	0	0	X	X	X	0	0	X	X	X	0	0
**Total**	X	X	X	0	0	X	X	X	0	0	X	X	X	0	0	X	X	X	0	0	X	X	X	0	0	X	X	X	0	0	X	X	X	0	0	X	X	X	0	0

Table S1 (Continuation)

Monitor Chart

Group: LPS (12 mg/ kg BW) + 50µg Fh15 (~ 2.5 mg/kg BW)

Date of injection with LPS- *E. coli*: May 13, 2021

Time of injection: 5:20 pm

Cage: #2 (n=5)

Variable	1h					2h					3h					4h					5h					6h					7h					8h					9h				
Appearance	0	0	0	0	0	0	0	0	0	0	0	0	0	0	0	0	0	0	0	0	0	0	0	0	0	0	0	0	0	0	0	0	0	0	0	1	1	1	1	1	1	1	1	1	1
Level of consciousness	0	0	0	0	0	0	0	0	0	0	0	0	0	0	0	0	0	0	0	0	0	0	0	0	0	0	0	0	0	0	0	0	0	0	0	0	0	0	0	0	0	1	0	1	1
Activity	0	0	0	0	0	0	0	0	0	0	0	0	0	0	0	0	0	0	0	0	0	0	0	0	0	0	0	0	0	0	0	0	0	0	0	0	1	0	0	0	1	2	1	1	1
Responses to stimulus	0	0	0	0	0	0	0	0	0	0	0	0	0	0	0	0	0	0	0	0	0	0	0	0	0	0	0	0	0	0	0	0	0	0	0	0	0	0	0	0	1	1	1	1	1
Eyes	0	0	0	0	0	0	0	0	0	0	0	0	0	0	0	0	0	0	0	0	0	0	0	0	0	0	0	0	0	0	0	0	0	0	0	1	0	1	1	1	1	1	1	1	1
Respiration rate	0	0	0	0	0	0	0	0	0	0	0	0	0	0	0	0	0	0	0	0	0	0	0	0	0	0	0	0	0	0	0	0	0	0	0		1	0	0	1	1	1	1	0	1
Respiration quality	0	0	0	0	0	0	0	0	0	0	0	0	0	0	0	0	0	0	0	0	0	0	0	0	0	0	0	0	0	0	0	0	0	0	0	0	0	0	0	0	0	0	0	0	0
**Total**	0	0	0	0	0	0	0	0	0	0	0	0	0	0	0	0	0	0	0	0	0	0	0	0	0	0	0	0	0	0	0	0	0	0	0	2	3	3	2	1	5	5	5	5	6

Table S1 (Continuation)
Monitor Chart
Group: LPS (12 mg/ kg BW) + 50μg Fh15 (~ 2.5 mg/kg BW)
Date of injection with LPS- *E. coli*: May 13, 2021
Time of injection: 5:20 pm
Cage: #2 (n=5)

Variable	10h					11h					12h					16h					20h					24h					28h					32h				
Appearance	2	2	2	2	2	2	2	2	2	2	3	3	3	2	3	3	3	4	3	3	4	4	4	4	4	2	X	X	4	2	1	X	X	X	1	0	X	X	X	0
Level of consciousness	1	1	1	1	1	2	2	2	2	2	2	2	2	2	2	2	3	3	3	3	3	4	4	3	3	1	X	X	3	1	0	X	X	X	0	0	X	X	X	0
Activity	1	2	2	1	1	2	2	2	2	2	2	2	3	2	2	3	3	3	3	3	4	4	4	3	3	2	X	X	4	2	1	X	X	X	0	0	X	X	X	0
Responses to stimulus	1	2	2	2	1	2	2	2	2	2	2	2	2	2	2	3	3	3	3	3	3	3	4	3	3	1	X	X	4	1	0	X	X	X	0	0	X	X	X	0
Eyes	2	2	2	2	2	2	2	2	2	2	2	3	2	2	2	3	3	3	3	3	3	4	4	3	3	2	X	X	4	2	1	X	X	X	0	0	X	X	X	0
Respiration rate	1	1	2	1	1	2	2	2	2	2	2	2	2	2	2	3	3	3	3	3	3	4	4	3	3	2	X	X	4	1	0	X	X	X	0	0	X	X	X	0
Respiration quality	1	1	1	0	1	1	2	2	1	1	2	2	2	2	2	2	2	3	2	2	3	3	3	3	3	1	X	X	3	1	0	X	X	X	0	0	X	X	X	0
**Total**	9	11	12	9	9	13	14	14	13	13	14	16	16	14	15	19	20	22	20	20	23	26	27	22	22	11	X	X	26	10	3	X	X	X	1	0	X	X	X	0

Table S1 (Continuation)
Monitor Chart
Group: LPS (12 mg/ kg BW) + 50µg Fh15 (~ 2.5 mg/kg BW)
Date of injection with LPS- *E. coli*: May 13, 2021
Time of injection: 5:20 pm
Cage: #2 (n=5)

Variable	36h					40h					44h					48					52h					56h					60h					72h				
Appearance	0	X	X	X	0	0	X	X	X	0	0	X	X	X	0	0	X	X	X	0	0	X	X	X	0	0	X	X	X	0	0	X	X	X	0	0	X	X	X	0
Level of consciousness	0	X	X	X	0	0	X	X	X	0	0	X	X	X	0	0	X	X	X	0	0	X	X	X	0	0	X	X	X	0	0	X	X	X	0	0	X	X	X	0
Activity	0	X	X	X	0	0	X	X	X	0	0	X	X	X	0	0	X	X	X	0	0	X	X	X	0	0	X	X	X	0	0	X	X	X	0	0	X	X	X	0
Responses to stimulus	0	X	X	X	0	0	X	X	X	0	0	X	X	X	0	0	X	X	X	0	0	X	X	X	0	0	X	X	X	0	0	X	X	X	0	0	X	X	X	0
Eyes	0	X	X	X	0	0	X	X	X	0	0	X	X	X	0	0	X	X	X	0	0	X	X	X	0	0	X	X	X	0	0	X	X	X	0	0	X	X	X	0
Respiration rate	0	X	X	X	0	0	X	X	X	0	0	X	X	X	0	0	X	X	X	0	0	X	X	X	0	0	X	X	X	0	0	X	X	X	0	0	X	X	X	0
Respiration quality	0	X	X	X	0	0	X	X	X	0	0	X	X	X	0	0	X	X	X	0	0	X	X	X	0	0	X	X	X	0	0	X	X	X	0	0	X	X	X	0
**Total**	0	X	X	X	0	0	X	X	X	0	0	X	X	X	0	0	X	X	X	0	0	X	X	X	0	0	X	X	X	0	0	X	X	X	0	0	X	X	X	0

Table S1 (Continuation)

Monitor Chart

Group: LPS (12 mg/ kg BW) + 50μg Fh15 (~2.5 mg/kg BW)

Date of injection with LPS- *E. coli*: December 15, 2022

Time of injection: 3:55 pm

Cage: #1 (n=5)

Variable	1h					2h					3h					4h					5h					6h					7h					8h					9h				
Appearance	0	0	0	0	0	0	0	0	0	0	0	0	0	0	0	0	0	0	0	0	0	0	0	0	0	0	0	0	0	0	0	0	0	0	0	1	1	1	1	1	1	1	1	1	1
Level of consciousness	0	0	0	0	0	0	0	0	0	0	0	0	0	0	0	0	0	0	0	0	0	0	0	0	0	0	0	0	0	0	0	0	0	0	0	0	0	0	0	0	0	0	1	0	1
Activity	0	0	0	0	0	0	0	0	0	0	0	0	0	0	0	0	0	0	0	0	0	0	0	0	0	0	0	0	0	0	0	0	0	0	0	0	0	0	0	0	1	1	1	1	1
Responses to stimulus	0	0	0	0	0	0	0	0	0	0	0	0	0	0	0	0	0	0	0	0	0	0	0	0	0	0	0	0	0	0	0	0	0	0	0	0	0	0	0	0	1	1	1	1	1
Eyes	0	0	0	0	0	0	0	0	0	0	0	0	0	0	0	0	0	0	0	0	0	0	0	0	0	0	0	0	0	0	0	0	0	0	0	1	1	1	0	0	1	1	1	1	1
Respiration rate	0	0	0	0	0	0	0	0	0	0	0	0	0	0	0	0	0	0	0	0	0	0	0	0	0	0	0	0	0	0	0	0	0	0	0	0	1	0	0	0	1	1	1	0	1
Respiration quality	0	0	0	0	0	0	0	0	0	0	0	0	0	0	0	0	0	0	0	0	0	0	0	0	0	0	0	0	0	0	0	0	0	0	0	0	0	0	0	0	0	0	0	0	1
**Total**	0	0	0	0	0	0	0	0	0	0	0	0	0	0	0	0	0	0	0	0	0	0	0	0	0	0	0	0	0	0	0	0	0	0	0	2	3	2	1	1	5	5	6	4	7

Table S1 (Continuation)

Monitor Chart

Group: LPS (12 mg/ kg BW) + 50µg Fh15 (~2.5 mg/kg BW)

Date of injection with LPS- *E. coli*: December 15, 2022

Time of injection: 3:55 pm

Cage: #1 (n=5)

Variable	10h					11h					12h					16h					20h					24h					28h					32h				
Appearance	2	2	2	2	2	2	2	2	2	2	3	3	3	2	3	3	3	3	3	3	4	4	4	4	4	2	X	X	2	2	1	X	X	1	1	0	X	X	0	0
Level of consciousness	1	1	1	1	1	1	2	2	1	2	2	2	2	2	2	3	3	3	3	3	3	4	3	3	3	1	X	X	2	1	0	X	X	0	0	0	X	X	0	0
Activity	1	1	2	1	1	2	2	2	2	2	2	2	3	2	2	3	3	3	3	3	4	4	4	4	4	2	X	X	2	2	0	X	X	0	0	0	X	X	0	0
Responses to stimulus	1	2	2	1	1	2	2	2	1	1	2	2	2	2	2	3	3	3	3	3	3	3	3	3	4	2	X	X	2	1	0	X	X	0	0	0	X	X	0	0
Eyes	2	2	2	1	2	2	2	2	2	2	2	2	3	2	2	3	3	3	3	3	4	4	4	4	4	2	X	X	2	2	0	X	X	1	0	0	X	X	0	0
Respiration rate	1	1	2	1	1	1	2	2	2	2	2	2	2	2	2	3	3	3	3	3	4	3	3	3	4	2	X	X	2	2	0	X	X	0	0	0	X	X	0	0
Respiration quality	0	1	1	0	1	1	1	2	1	1	1	2	2	2	2	3	3	2	2	2	3	3	3	3	3	1	X	X	1	1	0	X	X	0	0	0	X	X	0	0
**Total**	8	10	12	7	9	11	13	14	11	12	14	15	17	14	15	21	21	20	20	20	25	25	24	24	26	12	X	X	13	11	1	X	X	2	1	0	X	X	0	0

Table S1 (Continuation)
Monitor Chart
Group: LPS (12 mg/ kg BW) + 50µg Fh15 (~2.5 mg/kg BW)
Date of injection with LPS- *E. coli*: December 15, 2022
Time of injection: 3:55 pm
Cage: #1 (n=5)

Variable	36h					40h					44h					48					52h					56h					60h					72h				
Appearance	0	X	X	0	0	0	X	X	0	0	0	X	X	0	0	0	X	X	0	0	0	X	X	0	0	0	X	X	0	0	0	X	X	0	0	0	X	X	0	0
Level of consciousness	0	X	X	0	0	0	X	X	0	0	0	X	X	0	0	0	X	X	0	0	0	X	X	0	0	0	X	X	0	0	0	X	X	0	0	0	X	X	0	0
Activity	0	X	X	0	0	0	X	X	0	0	0	X	X	0	0	0	X	X	0	0	0	X	X	0	0	0	X	X	0	0	0	X	X	0	0	0	X	X	0	0
Responses to stimulus	0	X	X	0	0	0	X	X	0	0	0	X	X	0	0	0	X	X	0	0	0	X	X	0	0	0	X	X	0	0	0	X	X	0	0	0	X	X	0	0
Eyes	0	X	X	0	0	0	X	X	0	0	0	X	X	0	0	0	X	X	0	0	0	X	X	0	0	0	X	X	0	0	0	X	X	0	0	0	X	X	0	0
Respiration rate	0	X	X	0	0	0	X	X	0	0	0	X	X	0	0	0	X	X	0	0	0	X	X	0	0	0	X	X	0	0	0	X	X	0	0	0	X	X	0	0
Respiration quality	0	X	X	0	0	0	X	X	0	0	0	X	X	0	0	0	X	X	0	0	0	X	X	0	0	0	X	X	0	0	0	X	X	0	0	0	X	X	0	0
**Total**	0	X	X	0	0	0	X	X	0	0	0	X	X	0	0	0	X	X	0	0	0	X	X	0	0	0	X	X	0	0	0	X	X	0	0	0	X	X	0	0

Table S1 (Continuation)
Monitor Chart
Group: LPS (12 mg/ kg BW) + 100µg Fh15 (~ 5.0 mg/kg BW)
Date of injection with LPS- *E. coli*: May 13, 2021
Time of injection: 4:30 pm
Cage: #1 (n=5)

Variable	1h					2h					3h					4h					5h					6h					7h					8h					9h				
Appearance	0	0	0	0	0	0	0	0	0	0	0	0	0	0	0	0	0	0	0	0	0	0	0	0	0	0	0	0	0	0	0	0	0	0	0	1	1	1	1	1	1	1	1	1	1
Level of consciousness	0	0	0	0	0	0	0	0	0	0	0	0	0	0	0	0	0	0	0	0	0	0	0	0	0	0	0	0	0	0	0	0	0	0	0	0	0	0	0	0	0	1	0	1	0
Activity	0	0	0	0	0	0	0	0	0	0	0	0	0	0	0	0	0	0	0	0	0	0	0	0	0	0	0	0	0	0	0	0	0	0	0	0	0	1	0	0	1	1	1	1	1
Responses to stimulus	0	0	0	0	0	0	0	0	0	0	0	0	0	0	0	0	0	0	0	0	0	0	0	0	0	0	0	0	0	0	0	0	0	0	0	0	0	0	0	0	1	1	1	1	0
Eyes	0	0	0	0	0	0	0	0	0	0	0	0	0	0	0	0	0	0	0	0	0	0	0	0	0	0	0	0	0	0	0	0	0	0	0	1	1	1	1	1	1	1	1	1	1
Respiration rate	0	0	0	0	0	0	0	0	0	0	0	0	0	0	0	0	0	0	0	0	0	0	0	0	0	0	0	0	0	0	0	0	0	0	0	0	1	0	0	1	1	1	1	1	1
Respiration quality	0	0	0	0	0	0	0	0	0	0	0	0	0	0	0	0	0	0	0	0	0	0	0	0	0	0	0	0	0	0	0	0	0	0	0	0	0	0	0	0	0	0	0	0	0
**Total**	0	0	0	0	0	0	0	0	0	0	0	0	0	0	0	0	0	0	0	0	0	0	0	0	0	0	0	0	0	0	0	0	0	0	0	2	3	3	2	3	5	6	5	6	4

Table S1 (Continuation)
Monitor Chart
Group: LPS (12 mg/ kg BW) + 100µg Fh15 (~ 5.0 mg/kg BW)
Date of injection with LPS- *E. coli*: May 13, 2021
Time of injection: 4:30 pm
Cage: #1 (n=5)

Variable	10h					11h					12h					16h					20h					24h					28h					32h				
Appearance	1	2	2	2	2	2	2	2	2	2	2	3	2	2	2	3	3	2	2	3	3	4	3	3	3	4	X	3	3	4	X	X	3	2	3	X	X	0	0	1
Level of consciousness	1	1	1	1	1	1	1	1	2	1	1	2	1	2	1	2	2	2	2	2	2	3	2	2	2	3	X	3	3	3	X	X	2	1	1	X	X	0	0	0
Activity	1	1	1	1	1	2	2	2	2	1	2	2	2	2	2	2	3	2	2	3	3	4	3	3	3	3	X	4	3	4	X	X	2	2	3	X	X	1	1	1
Responses to stimulus	1	2	1	1	1	2	2	1	1	1	2	2	2	1	2	2	2	2	2	2	2	3	2	2	2	3	X	3	3	3	X	X	2	1	2	X	X	0	0	0
Eyes	2	2	1	2	1	2	2	2	2	2	2	2	2	2	2	2	2	2	2	2	3	3	3	2	3	3	X	3	3	3	X	X	2	2	3	X	X	0	1	1
Respiration rate	1	1	1	1	1	1	2	2	1	1	2	2	2	2	2	2	2	2	2	2	2	3	2	2	2	3	X	3	3	3	X	X	1	1	2	X	X	0	0	0
Respiration quality	0	1	0	0	0	1	1	1	1	1	1	2	1	1	1	1	2	2	1	1	2	3	2	1	2	3	X	3	3	3	X	X	1	1	1	X	X	0	0	0
**Total**	7	9	7	8	7	11	12	11	11	9	12	14	12	12	12	14	16	14	13	15	17	23	17	15	17	22	X	22	21	23	X	X	13	10	15	X	X	1	2	3

Table S1 (Continuation)
Monitor Chart
Group: LPS (12 mg/ kg BW) + 100µg Fh15 (~ 5.0 mg/kg BW)
Date of injection with LPS- *E. coli*: May 13, 2021
Time of injection: 4:30 pm
Cage: #1 (n=5)

Variable	36h					40h					44h					48					52h					56h					60h					72h				
Appearance	X	X	0	0	0	X	X	0	0	0	X	X	0	0	0	X	X	0	0	0	X	X	0	0	0	X	X	0	0	0	X	X	0	0	0	X	X	0	0	0
Level of consciousness	X	X	0	0	0	X	X	0	0	0	X	X	0	0	0	X	X	0	0	0	X	X	0	0	0	X	X	0	0	0	X	X	0	0	0	X	X	0	0	0
Activity	X	X	0	0	0	X	X	0	0	0	X	X	0	0	0	X	X	0	0	0	X	X	0	0	0	X	X	0	0	0	X	X	0	0	0	X	X	0	0	0
Responses to stimulus	X	X	0	0	0	X	X	0	0	0	X	X	0	0	0	X	X	0	0	0	X	X	0	0	0	X	X	0	0	0	X	X	0	0	0	X	X	0	0	0
Eyes	X	X	0	0	0	X	X	0	0	0	X	X	0	0	0	X	X	0	0	0	X	X	0	0	0	X	X	0	0	0	X	X	0	0	0	X	X	0	0	0
Respiration rate	X	X	0	0	0	X	X	0	0	0	X	X	0	0	0	X	X	0	0	0	X	X	0	0	0	X	X	0	0	0	X	X	0	0	0	X	X	0	0	0
Respiration quality	X	X	0	0	0	X	X	0	0	0	X	X	0	0	0	X	X	0	0	0	X	X	0	0	0	X	X	0	0	0	X	X	0	0	0	X	X	0	0	0
**Total**	X	X	0	0	0	X	X	0	0	0	X	X	0	0	0	X	X	0	0	0	X	X	0	0	0	X	X	0	0	0	X	X	0	0	0	X	X	0	0	0

Table S1 (Continuation)
Monitor Chart
Group: LPS (12 mg/ kg BW) + 100µg Fh15 (~ 5.0 mg/kg BW)
Date of injection with LPS- *E. coli*: May 13, 2021
Time of injection: 4:45 pm
Cage: #2 (n=5)

Variable	1h					2h					3h					4h					5h					6h					7h					8h					9h				
Appearance	0	0	0	0	0	0	0	0	0	0	0	0	0	0	0	0	0	0	0	0	0	0	0	0	0	0	0	0	0	0	0	0	0	0	0	1	1	1	1	1	1	1	1	1	1
Level of consciousness	0	0	0	0	0	0	0	0	0	0	0	0	0	0	0	0	0	0	0	0	0	0	0	0	0	0	0	0	0	0	0	0	0	0	0	0	0	0	0	0	0	0	0	0	0
Activity	0	0	0	0	0	0	0	0	0	0	0	0	0	0	0	0	0	0	0	0	0	0	0	0	0	0	0	0	0	0	0	0	0	0	0	0	0	0	1	0	1	1	1	1	1
Responses to stimulus	0	0	0	0	0	0	0	0	0	0	0	0	0	0	0	0	0	0	0	0	0	0	0	0	0	0	0	0	0	0	0	0	0	0	0	0	0	0	0	0	1	0	0	0	0
Eyes	0	0	0	0	0	0	0	0	0	0	0	0	0	0	0	0	0	0	0	0	0	0	0	0	0	0	0	0	0	0	0	0	0	0	0	1	0	1	0	0	1	1	1	1	1
Respiration rate	0	0	0	0	0	0	0	0	0	0	0	0	0	0	0	0	0	0	0	0	0	0	0	0	0	0	0	0	0	0	0	0	0	0	0	0	0	0	0	0	1	0	1	1	0
Respiration quality	0	0	0	0	0	0	0	0	0	0	0	0	0	0	0	0	0	0	0	0	0	0	0	0	0	0	0	0	0	0	0	0	0	0	0	0	0	0	0	0	0	0	0	0	0
**Total**	0	0	0	0	0	0	0	0	0	0	0	0	0	0	0	0	0	0	0	0	0	0	0	0	0	0	0	0	0	0	0	0	0	0	0	2	1	2	2	1	5	3	4	4	3

Table S1 (Continuation)

Monitor Chart

Group: LPS (12 mg/ kg BW) + 100µg Fh15 (~ 5.0 mg/kg BW)

Date of injection with LPS- *E. coli*: May 13, 2021

Time of injection: 4:45 pm

Cage: #2 (n=5)

Variable	10h					11h					12h					16h					20h					24h					28h					32h				
Appearance	2	1	2	2	1	2	2	2	2	1	3	2	2	2	2	3	2	2	3	2	3	3	3	3	3	4	3	3	4	4	3	2	2	3	2	1	1	0	1	1
Level of consciousness	1	1	1	1	0	1	1	1	1	1	2	1	2	1	1	2	2	2	2	2	2	2	2	2	3	3	3	3	3	3	2	1	1	2	2	0	1	0	0	0
Activity	1	1	1	1	1	2	1	2	2	2	2	2	2	2	2	3	3	3	2	3	3	3	3	3	3	3	3	3	4	4	2	2	2	2	2	1	1	1	1	1
Responses to stimulus	1	0	1	1	1	2	1	2	1	1	2	2	2	2	2	2	2	2	2	2	3	3	2	2	2	3	3	3	3	3	2	2	1	2	2	0	0	0	0	0
Eyes	2	1	1	1	1	2	2	2	2	2	2	2	2	2	2	3	2	2	2	3	3	3	3	3	3	3	3	3	4	4	2	2	1	2	3	1	1	0	0	1
Respiration rate	1	1	1	1	1	1	1	1	1	1	2	2	2	2	1	2	2	2	2	2	3	2	2	2	2	3	2	3	3	3	2	2	1	2	2	0	0	0	0	0
Respiration quality	0	0	0	0	0	1	0	1	0	0	1	1	2	1	1	2	2	2	2	2	2	2	2	2	2	2	2	2	2	3	1	1	1	2	2	0	0	0	0	0
**Total**	8	5	7	7	5	11	8	11	9	8	14	12	14	12	11	17	15	15	15	16	19	18	17	17	18	21	19	20	23	24	14	12	9	15	15	3	4	1	2	3

Table S1 (Continuation)
Monitor Chart
Group: LPS (12 mg/ kg BW) + 100µg Fh15 (~ 5.0 mg/kg BW)
Date of injection with LPS- *E. coli*: May 13, 2021
Time of injection: 4:45 pm
Cage: #2 (n=5)

Variable	36h					40h					44h					48					52h					56h					60h					72h				
Appearance	0	0	0	0	0	0	0	0	0	0	0	0	0	0	0	0	0	0	0	0	0	0	0	0	0	0	0	0	0	0	0	0	0	0	0	0	0	0	0	0
Level of consciousness	0	0	0	0	0	0	0	0	0	0	0	0	0	0	0	0	0	0	0	0	0	0	0	0	0	0	0	0	0	0	0	0	0	0	0	0	0	0	0	0
Activity	0	0	0	0	0	0	0	0	0	0	0	0	0	0	0	0	0	0	0	0	0	0	0	0	0	0	0	0	0	0	0	0	0	0	0	0	0	0	0	0
Responses to stimulus	0	0	0	0	0	0	0	0	0	0	0	0	0	0	0	0	0	0	0	0	0	0	0	0	0	0	0	0	0	0	0	0	0	0	0	0	0	0	0	0
Eyes	0	0	0	0	0	0	0	0	0	0	0	0	0	0	0	0	0	0	0	0	0	0	0	0	0	0	0	0	0	0	0	0	0	0	0	0	0	0	0	0
Respiration rate	0	0	0	0	0	0	0	0	0	0	0	0	0	0	0	0	0	0	0	0	0	0	0	0	0	0	0	0	0	0	0	0	0	0	0	0	0	0	0	0
Respiration quality	0	0	0	0	0	0	0	0	0	0	0	0	0	0	0	0	0	0	0	0	0	0	0	0	0	0	0	0	0	0	0	0	0	0	0	0	0	0	0	0
**Total**	0	0	0	0	0	0	0	0	0	0	0	0	0	0	0	0	0	0	0	0	0	0	0	0	0	0	0	0	0	0	0	0	0	0	0	0	0	0	0	0

Table S1 (Continuation)

Monitor Chart

Group: LPS (12 mg/ kg BW) + 100µg Fh15 (~ 5.0 mg/kg BW)

Date of injection with LPS- *E. coli*: December 15, 2022

Time of injection: 4:10 pm

Cage: #1 (n=5)

Variable	1h					2h					3h					4h					5h					6h					7h					8h					9h				
Appearance	0	0	0	0	0	0	0	0	0	0	0	0	0	0	0	0	0	0	0	0	0	0	0	0	0	0	0	0	0	0	0	0	0	0	0	1	1	1	1	1	1	1	1	1	1
Level of consciousness	0	0	0	0	0	0	0	0	0	0	0	0	0	0	0	0	0	0	0	0	0	0	0	0	0	0	0	0	0	0	0	0	0	0	0	0	0	0	0	0	0	1	0	0	0
Activity	0	0	0	0	0	0	0	0	0	0	0	0	0	0	0	0	0	0	0	0	0	0	0	0	0	0	0	0	0	0	0	0	0	0	0	0	0	0	0	0	1	1	1	1	1
Responses to stimulus	0	0	0	0	0	0	0	0	0	0	0	0	0	0	0	0	0	0	0	0	0	0	0	0	0	0	0	0	0	0	0	0	0	0	0	0	0	0	0	0	1	1	0	1	1
Eyes	0	0	0	0	0	0	0	0	0	0	0	0	0	0	0	0	0	0	0	0	0	0	0	0	0	0	0	0	0	0	0	0	0	0	0	1	0	0	0	0	1	1	1	1	1
Respiration rate	0	0	0	0	0	0	0	0	0	0	0	0	0	0	0	0	0	0	0	0	0	0	0	0	0	0	0	0	0	0	0	0	0	0	0	0	0	0	0	0	1	1	0	0	1
Respiration quality	0	0	0	0	0	0	0	0	0	0	0	0	0	0	0	0	0	0	0	0	0	0	0	0	0	0	0	0	0	0	0	0	0	0	0	0	0	0	0	0	0	0	0	0	0
**Total**	0	0	0	0	0	0	0	0	0	0	0	0	0	0	0	0	0	0	0	0	0	0	0	0	0	0	0	0	0	0	0	0	0	0	0	2	1	1	1	1	5	6	3	4	5

Table S1 (Continuation)

Monitor Chart

Group: LPS (12 mg/ kg BW) + 100µg Fh15 (~ 5.0 mg/kg BW)

Date of injection with LPS- *E. coli*: December 15, 2022

Time of injection: 4:10 pm

Cage: #1 (n=5)

Variable	10h					11h					12h					16h					20h					24h					28h					32h				
Appearance	1	2	1	1	2	2	2	2	2	2	2	3	2	3	2	2	3	3	3	3	3	3	3	3	3	4	4	4	4	4	2	2	3	2	3	1	1	1	1	1
Level of consciousness	1	1	1	1	1	2	2	1	1	1	2	2	2	2	2	2	2	3	2	2	2	2	2	3	2	3	3	3	3	3	1	1	2	1	1	0	0	0	0	1
Activity	1	2	0	1	1	2	2	1	2	2	2	2	2	2	2	2	3	2	2	2	2	3	3	2	3	3	4	4	3	3	2	2	2	2	2	0	0	1	1	1
Responses to stimulus	1	1	1	1	1	1	2	1	2	2	2	2	2	2	2	2	2	2	2	3	3	3	2	2	3	3	3	3	3	3	1	2	2	1	2	0	0	0	0	0
Eyes	1	1	1	1	1	2	2	1	2	2	2	2	2	2	2	2	2	3	2	3	3	3	3	3	3	3	3	4	3	3	2	2	2	2	2	1	0	0	0	1
Respiration rate	1	1	1	1	1	1	1	1	1	1	1	2	1	2	2	2	2	2	2	2	2	3	3	2	3	3	3	4	3	3	1	2	2	1	2	0	0	0	0	0
Respiration quality	1	1	0	1	1	1	1	1	1	1	1	1	1	1	2	2	2	2	2	2	2	2	2	2	2	3	3	3	2	2	1	1	2	0	1	0	0	0	0	0
**Total**	7	9	5	7	8	11	12	8	11	11	12	14	12	14	14	14	16	17	15	17	17	19	18	17	19	22	23	25	21	21	10	12	15	9	13	2	1	2	2	4

Table S1 (Continuation)
Monitor Chart
Group: LPS (12 mg/ kg BW) + 100µg Fh15 (~ 5.0 mg/kg BW)
Date of injection with LPS- *E. coli*: December 15, 2022
Time of injection: 4:10 pm
Cage: #1 (n=5)

Variable	36h					40h					44h					48					52h					56h					60h					72h				
Appearance	0	0	0	0	0	0	0	0	0	0	0	0	0	0	0	0	0	0	0	0	0	0	0	0	0	0	0	0	0	0	0	0	0	0	0	0	0	0	0	0
Level of consciousness	0	0	0	0	0	0	0	0	0	0	0	0	0	0	0	0	0	0	0	0	0	0	0	0	0	0	0	0	0	0	0	0	0	0	0	0	0	0	0	0
Activity	0	0	0	0	0	0	0	0	0	0	0	0	0	0	0	0	0	0	0	0	0	0	0	0	0	0	0	0	0	0	0	0	0	0	0	0	0	0	0	0
Responses to stimulus	0	0	0	0	0	0	0	0	0	0	0	0	0	0	0	0	0	0	0	0	0	0	0	0	0	0	0	0	0	0	0	0	0	0	0	0	0	0	0	0
Eyes	0	0	0	0	0	0	0	0	0	0	0	0	0	0	0	0	0	0	0	0	0	0	0	0	0	0	0	0	0	0	0	0	0	0	0	0	0	0	0	0
Respiration rate	0	0	0	0	0	0	0	0	0	0	0	0	0	0	0	0	0	0	0	0	0	0	0	0	0	0	0	0	0	0	0	0	0	0	0	0	0	0	0	0
Respiration quality	0	0	0	0	0	0	0	0	0	0	0	0	0	0	0	0	0	0	0	0	0	0	0	0	0	0	0	0	0	0	0	0	0	0	0	0	0	0	0	0
**Total**	0	0	0	0	0	0	0	0	0	0	0	0	0	0	0	0	0	0	0	0	0	0	0	0	0	0	0	0	0	0	0	0	0	0	0	0	0	0	0	0

Table S1 (Continuation)

Monitor Chart

Group: LPS (12 mg/ kg BW) + 200µg Fh15 (~ 10.0 mg/kg BW)

Date of injection with LPS- *E. coli*: May 13, 2021

Time of injection: 12:20 pm

Cage: #1 (n=5)

Variable	1h					2h					3h					4h					5h					6h					7h					8h					9h				
Appearance	0	0	0	0	0	0	0	0	0	0	0	0	0	0	0	0	0	0	0	0	0	0	0	0	0	0	0	0	0	0	0	0	0	0	0	1	1	1	1	1	1	2	2	1	1
Level of consciousness	0	0	0	0	0	0	0	0	0	0	0	0	0	0	0	0	0	0	0	0	0	0	0	0	0	0	0	0	0	0	0	0	0	0	0	0	0	0	0	0	1	1	1	1	1
Activity	0	0	0	0	0	0	0	0	0	0	0	0	0	0	0	0	0	0	0	0	0	0	0	0	0	0	0	0	0	0	0	0	0	0	0	1	1	0	1	1	1	1	1	1	1
Responses to stimulus	0	0	0	0	0	0	0	0	0	0	0	0	0	0	0	0	0	0	0	0	0	0	0	0	0	0	0	0	0	0	0	0	0	0	0	0	0	0	0	0	1	1	1	1	1
Eyes	0	0	0	0	0	0	0	0	0	0	0	0	0	0	0	0	0	0	0	0	0	0	0	0	0	0	0	0	0	0	0	0	0	0	0	1	1	1	1	1	2	1	1	1	1
Respiration rate	0	0	0	0	0	0	0	0	0	0	0	0	0	0	0	0	0	0	0	0	0	0	0	0	0	0	0	0	0	0	0	0	0	0	0	0	0	0	0	0	1	1	1	1	1
Respiration quality	0	0	0	0	0	0	0	0	0	0	0	0	0	0	0	0	0	0	0	0	0	0	0	0	0	0	0	0	0	0	0	0	0	0	0	0	0	0	0	0	0	0	1	0	0
**Total**	0	0	0	0	0	0	0	0	0	0	0	0	0	0	0	0	0	0	0	0	0	0	0	0	0	0	0	0	0	0	0	0	0	0	0	3	3	2	3	3	7	7	8	6	6

Table S1 (Continuation)
Monitor Chart
Group: LPS (12 mg/ kg BW) + 200µg Fh15 (~ 10.0 mg/kg BW)
Date of injection with LPS- *E. coli*: May 13, 2021
Time of injection: 12:20 pm
Cage: #1 (n=5)

Variable	10h					11h					12h					16h					20h					24h					28h					32h				
Appearance	2	2	2	2	2	3	2	2	2	2	3	3	2	3	2	3	3	3	3	3	4	3	4	3	3	4	4	4	4	3	X	X	X	X	3	X	X	X	X	1
Level of consciousness	2	1	1	1	2	2	2	1	2	2	3	2	2	2	2	3	3	2	2	2	3	3	3	3	3	4	4	4	3	3	X	X	X	X	2	X	X	X	X	1
Activity	2	2	2	2	2	2	2	2	2	2	3	3	2	2	3	3	3	2	3	3	3	4	3	3	3	4	4	4	4	3	X	X	X	X	3	X	X	X	X	1
Responses to stimulus	2	2	1	1	1	2	2	1	1	1	2	2	2	1	2	2	2	2	2	2	3	3	3	3	2	4	4	4	3	3	X	X	X	X	2	X	X	X	X	1
Eyes	2	2	2	2	1	2	2	2	2	2	3	3	2	2	2	3	3	2	3	3	3	3	3	3	3	4	4	4	4	3	X	X	X	X	3	X	X	X	X	1
Respiration rate	2	2	1	1	1	2	2	2	2	2	2	2	2	2	2	2	2	2	2	2	3	3	3	3	2	4	4	4	4	3	X	X	X	X	2	X	X	X	X	1
Respiration quality	1	1	1	1	1	1	1	1	1	1	1	1	1	1	1	2	1	2	1	1	2	3	3	2	2	4	4	4	3	2	X	X	X	X	2	X	X	X	X	1
**Total**	13	12	10	10	10	14	13	11	12	12	17	16	13	14	14	18	17	15	16	16	21	22	22	20	18	28	28	28	25	20	X	X	X	X	17	X	X	X	X	7

Table S1 (Continuation)
Monitor Chart
Group: LPS (12 mg/ kg BW) + 200µg Fh15 (~ 10.0 mg/kg BW)
Date of injection with LPS- *E. coli*: May 13, 2021
Time of injection: 12:20 pm
Cage: #1 (n=5)

Variable	36h					40h					44h					48					52h					56h					60h					72h				
Appearance	X	X	X	X	1	X	X	X	X	0	X	X	X	X	0	X	X	X	X	0	X	X	X	X	0	X	X	X	X	0	X	X	X	X	0	X	X	X	X	0
Level of consciousness	X	X	X	X	0	X	X	X	X	0	X	X	X	X	0	X	X	X	X	0	X	X	X	X	0	X	X	X	X	0	X	X	X	X	0	X	X	X	X	0
Activity	X	X	X	X	0	X	X	X	X	0	X	X	X	X	0	X	X	X	X	0	X	X	X	X	0	X	X	X	X	0	X	X	X	X	0	X	X	X	X	0
Responses to stimulus	X	X	X	X	0	X	X	X	X	0	X	X	X	X	0	X	X	X	X	0	X	X	X	X	0	X	X	X	X	0	X	X	X	X	0	X	X	X	X	0
Eyes	X	X	X	X	1	X	X	X	X	0	X	X	X	X	0	X	X	X	X	0	X	X	X	X	0	X	X	X	X	0	X	X	X	X	0	X	X	X	X	0
Respiration rate	X	X	X	X	1	X	X	X	X	0	X	X	X	X	0	X	X	X	X	0	X	X	X	X	0	X	X	X	X	0	X	X	X	X	0	X	X	X	X	0
Respiration quality	X	X	X	X	0	X	X	X	X	0	X	X	X	X	0	X	X	X	X	0	X	X	X	X	0	X	X	X	X	0	X	X	X	X	0	X	X	X	X	0
**Total**	X	X	X	X	3	X	X	X	X	0	X	X	X	X	0	X	X	X	X	0	X	X	X	X	0	X	X	X	X	0	X	X	X	X	0	X	X	X	X	0

Table S1 (Continuation)
Monitor Chart
Group: LPS (12 mg/ kg BW) + 200μg Fh15 (~ 10.0 mg/kg BW)
Date of injection with LPS- *E. coli*: May 13, 2021
Time of injection: 12:35 pm
Cage: #2 (n=5)

Variable	1h					2h					3h					4h					5h					6h					7h					8h					9h				
Appearance	0	0	0	0	0	0	0	0	0	0	0	0	0	0	0	0	0	0	0	0	0	0	0	0	0	0	0	0	0	0	0	0	0	0	0	1	1	1	1	1	1	1	1	1	1
Level of consciousness	0	0	0	0	0	0	0	0	0	0	0	0	0	0	0	0	0	0	0	0	0	0	0	0	0	0	0	0	0	0	0	0	0	0	0	0	0	0	0	0	1	1	1	0	0
Activity	0	0	0	0	0	0	0	0	0	0	0	0	0	0	0	0	0	0	0	0	0	0	0	0	0	0	0	0	0	0	0	0	0	0	0	1	1	1	1	1	1	1	1	1	1
Responses to stimulus	0	0	0	0	0	0	0	0	0	0	0	0	0	0	0	0	0	0	0	0	0	0	0	0	0	0	0	0	0	0	0	0	0	0	0	0	0	0	0	0	1	0	1	1	1
Eyes	0	0	0	0	0	0	0	0	0	0	0	0	0	0	0	0	0	0	0	0	0	0	0	0	0	0	0	0	0	0	0	0	0	0	0	1	0	1	0	1	1	1	1	1	1
Respiration rate	0	0	0	0	0	0	0	0	0	0	0	0	0	0	0	0	0	0	0	0	0	0	0	0	0	0	0	0	0	0	0	0	0	0	0	0	0	0	0	0	1	1	1	1	1
Respiration quality	0	0	0	0	0	0	0	0	0	0	0	0	0	0	0	0	0	0	0	0	0	0	0	0	0	0	0	0	0	0	0	0	0	0	0	0	0	0	0	0	0	0	0	0	0
**Total**	0	0	0	0	0	0	0	0	0	0	0	0	0	0	0	0	0	0	0	0	0	0	0	0	0	0	0	0	0	0	0	0	0	0	0	3	2	3	2	3	6	5	6	5	5

Table S1 (Continuation)
Monitor Chart
Group: LPS (12 mg/ kg BW) + 200µg Fh15 (~ 10.0 mg/kg BW)
Date of injection with LPS- *E. coli*: May 13, 2021
Time of injection: 12:35 pm
Cage: #2 (n=5)

Variable	10h					11h					12h					16h					20h					24h					28h					32h				
Appearance	2	1	2	1	2	2	2	2	2	2	3	2	3	3	3	3	3	3	3	3	4	4	3	3	4	4	4	4	4	3	X	X	2	2	3	X	X	1	2	1
Level of consciousness	1	1	1	1	1	2	2	1	1	2	2	2	2	1	2	2	3	3	3	3	3	3	3	3	3	4	3	3	3	2	X	X	2	1	2	X	X	1	1	1
Activity	2	2	1	2	2	2	2	2	2	2	3	2	3	3	2	3	3	3	3	3	4	4	3	3	4	4	4	4	4	3	X	X	3	2	2	X	X	1	1	1
Responses to stimulus	1	1	2	1	1	2	2	2	2	2	2	2	2	2	2	3	3	2	3	3	3	3	3	3	3	3	4	3	3	2	X	X	2	2	2	X	X	1	1	1
Eyes	2	2	2	2	2	2	2	2	3	2	3	3	2	2	3	3	3	3	3	3	3	3	3	3	4	4	4	3	3	3	X	X	3	2	2	X	X	1	1	1
Respiration rate	1	1	1	1	1	1	2	2	2	2	2	2	2	2	2	3	3	3	3	3	3	3	3	3	4	4	4	3	3	3	X	X	2	2	2	X	X	1	1	1
Respiration quality	1	1	1	1	1	1	1	1	1	1	1	1	2	1	1	2	2	2	2	3	3	2	2	3	3	3	3	3	2	2	X	X	2	2	1	X	X	1	1	0
**Total**	10	9	10	9	10	12	13	12	13	13	16	14	16	14	15	19	20	19	20	21	23	22	20	21	25	26	26	23	22	18	X	X	16	13	14	X	X	7	8	6

Table S1 (Continuation)
Monitor Chart
Group: LPS (12 mg/ kg BW) + 200µg Fh15 (~ 10.0 mg/kg BW)
Date of injection with LPS- *E. coli*: May 13, 2021
Time of injection: 12:35 pm
Cage: #2 (n=5)

Variable	36h					40h					44h					48					52h					56h					60h					72h				
Appearance	X	X	1	1	1	X	X	0	0	0	X	X	0	0	0	X	X	0	0	0	X	X	0	0	0	X	X	0	0	0	X	X	0	0	0	X	X	0	0	0
Level of consciousness	X	X	0	0	0	X	X	0	0	0	X	X	0	0	0	X	X	0	0	0	X	X	0	0	0	X	X	0	0	0	X	X	0	0	0	X	X	0	0	0
Activity	X	X	1	1	1	X	X	0	0	0	X	X	0	0	0	X	X	0	0	0	X	X	0	0	0	X	X	0	0	0	X	X	0	0	0	X	X	0	0	0
Responses to stimulus	X	X	0	0	0	X	X	0	0	0	X	X	0	0	0	X	X	0	0	0	X	X	0	0	0	X	X	0	0	0	X	X	0	0	0	X	X	0	0	0
Eyes	X	X	0	0	1	X	X	0	0	0	X	X	0	0	0	X	X	0	0	0	X	X	0	0	0	X	X	0	0	0	X	X	0	0	0	X	X	0	0	0
Respiration rate	X	X	0	1	0	X	X	0	0	0	X	X	0	0	0	X	X	0	0	0	X	X	0	0	0	X	X	0	0	0	X	X	0	0	0	X	X	0	0	0
Respiration quality	X	X	0	0	0	X	X	0	0	0	X	X	0	0	0	X	X	0	0	0	X	X	0	0	0	X	X	0	0	0	X	X	0	0	0	X	X	0	0	0
**Total**	X	X	2	3	3	X	X	0	0	0	X	X	0	0	0	X	X	0	0	0	X	X	0	0	0	X	X	0	0	0	X	X	0	0	0	X	X	0	0	0

Table S1 (Continuation)
Monitor Chart
Group: LPS (12 mg/ kg BW) + 200μg Fh15 (~ 10.0 mg/kg BW)
Date of injection with LPS- *E. coli*: December 15, 2022
Time of injection: 2:30 pm
Cage: #1 (n=5)

Variable	1h					2h					3h					4h					5h					6h					7h					8h					9h				
Appearance	0	0	0	0	0	0	0	0	0	0	0	0	0	0	0	0	0	0	0	0	0	0	0	0	0	0	0	0	0	0	0	0	0	0	0	1	1	1	1	1	2	2	2	1	1
Level of consciousness	0	0	0	0	0	0	0	0	0	0	0	0	0	0	0	0	0	0	0	0	0	0	0	0	0	0	0	0	0	0	0	0	0	0	0	0	0	0	0	0	1	1	1	1	0
Activity	0	0	0	0	0	0	0	0	0	0	0	0	0	0	0	0	0	0	0	0	0	0	0	0	0	0	0	0	0	0	0	0	0	0	0	0	1	1	1	1	1	1	1	1	1
Responses to stimulus	0	0	0	0	0	0	0	0	0	0	0	0	0	0	0	0	0	0	0	0	0	0	0	0	0	0	0	0	0	0	0	0	0	0	0	0	0	0	0	0	1	1	1	1	1
Eyes	0	0	0	0	0	0	0	0	0	0	0	0	0	0	0	0	0	0	0	0	0	0	0	0	0	0	0	0	0	0	0	0	0	0	0	0	0	0	0	1	1	1	1	1	1
Respiration rate	0	0	0	0	0	0	0	0	0	0	0	0	0	0	0	0	0	0	0	0	0	0	0	0	0	0	0	0	0	0	0	0	0	0	0	0	0	0	0	0	1	1	1	1	1
Respiration quality	0	0	0	0	0	0	0	0	0	0	0	0	0	0	0	0	0	0	0	0	0	0	0	0	0	0	0	0	0	0	0	0	0	0	0	0	0	0	0	0	0	0	0	0	0
**Total**	0	0	0	0	0	0	0	0	0	0	0	0	0	0	0	0	0	0	0	0	0	0	0	0	0	0	0	0	0	0	0	0	0	0	0	1	2	2	2	3	7	7	7	6	5

Table S1 (Continuation)

Monitor Chart

Group: LPS (12 mg/ kg BW) + 200µg Fh15 (~ 10.0 mg/kg BW)

Date of injection with LPS- *E. coli*: December 15, 2022

Time of injection: 2:30 pm

Cage: #1 (n=5)

Variable	10h					11h					12h					16h					20h					24h					28h					32h				
Appearance	1	2	1	1	2	2	2	2	2	2	2	3	2	3	2	2	3	3	3	3	3	3	3	3	3	4	4	4	4	4	2	2	3	2	3	X	X	X	1	1
Level of consciousness	1	1	1	1	1	2	2	1	1	1	2	2	2	2	2	2	2	3	2	2	2	2	2	3	2	3	3	3	3	3	1	1	2	1	1	X	X	X	0	1
Activity	1	2	0	1	1	2	2	1	2	2	2	2	2	2	2	2	3	2	2	2	2	3	3	2	3	3	4	4	3	3	2	2	2	2	2	X	X	X	1	1
Responses to stimulus	1	1	1	1	1	1	2	1	2	2	2	2	2	2	2	2	2	2	2	3	3	3	2	2	3	3	3	3	3	3	1	2	2	1	2	X	X	X	0	0
Eyes	1	1	1	1	1	2	2	1	2	2	2	2	2	2	2	2	2	3	2	3	3	3	3	3	3	3	3	4	3	3	2	2	2	2	2	X	X	X	0	1
Respiration rate	1	1	1	1	1	1	1	1	1	1	1	2	1	2	2	2	2	2	2	2	2	3	3	2	3	3	3	4	3	3	1	2	2	1	2	X	X	X	0	0
Respiration quality	1	1	0	1	1	1	1	1	1	1	1	1	1	1	2	2	2	2	2	2	2	2	2	2	2	3	3	3	2	2	1	1	2	0	1	X	X	X	0	0
**Total**	10	9	12	12	11	12	11	14	14	14	16	13	17	17	16	13	17	19	20	20	14	21	25	24	26	19	24	26	20	19	28	28	X	16	14	X	X	X	6	5

Table S1 (Continuation)

Monitor Chart

Group: LPS (12 mg/ kg BW) + 200µg Fh15 (~ 10.0 mg/kg BW)

Date of injection with LPS- *E. coli*: December 15, 2022

Time of injection: 2:30 pm

Cage: #1 (n=5)

Variable	36h					40h					44h					48					52h					56h					60h					72h				
Appearance	X	X	X	1	1	X	X	X	0	0	X	X	X	0	0	X	X	X	0	0	X	X	X	0	0	X	X	X	0	0	X	X	X	0	0	X	X	X	0	0
Level of consciousness	X	X	X	0	0	X	X	X	0	0	X	X	X	0	0	X	X	X	0	0	X	X	X	0	0	X	X	X	0	0	X	X	X	0	0	X	X	X	0	0
Activity	X	X	X	1	1	X	X	X	0	0	X	X	X	0	0	X	X	X	0	0	X	X	X	0	0	X	X	X	0	0	X	X	X	0	0	X	X	X	0	0
Responses to stimulus	X	X	X	0	0	X	X	X	0	0	X	X	X	0	0	X	X	X	0	0	X	X	X	0	0	X	X	X	0	0	X	X	X	0	0	X	X	X	0	0
Eyes	X	X	X	0	0	X	X	X	0	0	X	X	X	0	0	X	X	X	0	0	X	X	X	0	0	X	X	X	0	0	X	X	X	0	0	X	X	X	0	0
Respiration rate	X	X	X	0	0	X	X	X	0	0	X	X	X	0	0	X	X	X	0	0	X	X	X	0	0	X	X	X	0	0	X	X	X	0	0	X	X	X	0	0
Respiration quality	X	X	X	0	0	X	X	X	0	0	X	X	X	0	0	X	X	X	0	0	X	X	X	0	0	X	X	X	0	0	X	X	X	0	0	X	X	X	0	0
**Total**	X	X	X	2	2	X	X	X	0	0	X	X	X	0	0	X	X	X	0	0	X	X	X	0	0	X	X	X	0	0	X	X	X	0	0	X	X	X	0	0
